# HaploRec: efficient and accurate large-scale reconstruction of haplotypes

**DOI:** 10.1186/1471-2105-7-542

**Published:** 2006-12-22

**Authors:** Lauri Eronen, Floris Geerts, Hannu Toivonen

**Affiliations:** 1 HIIT-BRU, Department of Computer Science, University of Helsinki, Finland; 2 Laboratory for Foundations of Computer Science, University of Edinburgh, UK; 3 Department of Computer Science, University of Freiburg, Germany

## Abstract

**Background:**

Haplotypes extracted from human DNA can be used for gene mapping and other analysis of genetic patterns within and across populations. A fundamental problem is, however, that current practical laboratory methods do not give haplotype information. Estimation of phased haplotypes of unrelated individuals given their unphased genotypes is known as the haplotype reconstruction or phasing problem.

**Results:**

We define three novel statistical models and give an efficient algorithm for haplotype reconstruction, jointly called HaploRec. HaploRec is based on exploiting local regularities conserved in haplotypes: it reconstructs haplotypes so that they have maximal local coherence. This approach – not assuming statistical dependence for remotely located markers – has two useful properties: it is well-suited for sparse marker maps, such as those used in gene mapping, and it can actually take advantage of long maps.

**Conclusion:**

Our experimental results with simulated and real data show that HaploRec is a powerful method for the large scale haplotyping needed in association studies. With sample sizes large enough for gene mapping it appeared to be the best compared to all other tested methods (Phase, fastPhase, PL-EM, Snphap, Gerbil; simulated data), with small samples it was competitive with the best available methods (real data). HaploRec is several orders of magnitude faster than Phase and comparable to the other methods; the running times are roughly linear in the number of subjects and the number of markers. HaploRec is publicly available at .

## Background

The problem we consider is *haplotype reconstruction*: given the genotypes of a sample of individuals, the task is to predict the most likely haplotype pair for each individual. Computational haplotype reconstruction methods are based on statistical dependency between closely located markers, known as *linkage disequilibrium*. Many computational methods have been developed for the reconstruction of haplotypes. Some of these methods do not rely on the statistical modeling of the haplotypes [1-3], but most of them, like our proposed algorithm HaploRec, do [4-10]. For a review of these and other haplotyping methods we refer to [11-13]. Laboratory techniques are being developed for direct molecular haplotyping (see, e.g., [14,15]), but these techniques are not mature yet, and are currently time consuming and expensive.

The need for a new haplotyping method is motivated by high throughput association analysis, where the goal is to locate a disease susceptibility gene by finding a haplotype fragment that is associated with the disease being studied. More and more often gene mapping studies use a large marker map spread over a long genomic region. A typical strategy for computationally haplotyping a long map is to first divide the map to small, overlapping windows, to reconstruct the haplotypes in each window separately, and then to combine haplotypes from the windows [16]. HaploRec is aimed to have the following important properties. First, increasing the window size should give relatively more accurate results since large windows contain more information, i.e., adding markers should improve accuracy (in the phases between the markers that already were there), whether the new markers are added between the old ones or not [17]. Second, the time complexity of the algorithm should be close to linear in the number of markers, in order to avoid unnecessary compromises when choosing the window size, and also close to linear in the number of genotypes to allow sample sizes of hundreds to thousands of individuals, as required by association analysis [18]. HaploRec produces accurate haplotype reconstructions, and scales to long marker maps (or windows) that span long genetic regions.

While the statistical models of HaploRec are novel, some of the algorithmic principles are similar to earlier work. HaploRec follows a likelihood-based expectation-maximization (EM) haplotype inference strategy which was introduced in [4]. PL-EM [5,6] overcomes the computational complexity of the basic EM approach by using a a pruning strategy on the possible haplotype resolutions, called Partition-Ligation (PL). The Snphap algorithm of Clayton [19] is also based on the EM algorithm, but uses a sequential pruning strategy; HaploRec also uses a similar pruning approach. The PL strategy is also used in the current version of Phase [7,8]. PL-EM and Snphap are based on a multinomial haplotype probability model with a uniform Dirichlet prior. Phase, however, uses a prior distribution based on coalescent theory (see [20] for a review) and uses Bayesian inference implemented with Gibbs sampling instead of EM. The underlying idea for our statistical models for haplotypes is that we derive an overall probability for a haplotype from the probabilities of its local fragments. We propose three probability models based on this idea: high-order Markov chains, variable-order Markov chains, and a segmentation-based model. Moreover, a novelty is that the lengths of the haplotype fragments utilized in the models vary, governed by a *frequency threshold*. In contrast to most other approaches, long-range dependencies between markers are not required for the methods to work, but they can be utilized where they do exist. Thus all these models scale naturally to the long and sparse marker maps often used in gene mapping.

Our segmentation-based model bears some resemblance to methods which combine haplotype block finding and haplotyping [9,10]. However, whereas these models place universal block boundaries across the whole population, our model averages over all possible segmentations for each haplotype, without any fixed block boundaries.

Quite recently, Scheet and Stephens [21] introduced fastPhase, which models the population with a set of founder haplotypes (or clusters); the cluster memberships are allowed to change continuously along the chromosome, according to a hidden Markov model. Similar to our models, fastPhase allows for both "block-like" patterns and gradual decline of linkage disequilibrium with distance.

In our extensive experimental evaluation in the Results section we will compare HaploRec with Phase, fastPhase, PL-EM, Snphap, and Gerbil [10]. In the simulated settings, where sample sizes (number of subjects) are large enough for gene mapping, we observe the concrete benefits of the ability of HaploRec to improve its performance by haplotyping more markers at a time. The models we describe are relatively simple and are slightly outperformed by Phase when the number of markers is small, but when our method is given a longer map to be haplotyped, it can actually utilize the information contained in the additional markers to outperform Phase. HaploRec is in practice several orders of magnitude faster than Phase and has running times comparable to the other methods.

We next define the necessary notation.

### Notation and problem statement

We assume a set (map, or window) *M *of ℓ markers 1,...,ℓ and denote the set of all observed alleles of marker *i *by *A*_*i*_. A *haplotype H *over *M *is then a vector of alleles: *H *∈ Π_*i *= 1,...,ℓ _*A*_*i*_. A *genotype G *over *M *is a vector of (unordered) allele pairs: *G *∈ Π_*i *= 1,...,ℓ_{{*a*_1_, *a*_2_} | *a*_1_, *a*_2 _∈ *A*_*i*_}. For SNP markers, |*A*_*i*_| = 2. Assuming that alleles are labeled "1" and "2", SNP haplotypes are vectors in {1, 2}^ℓ ^and SNP genotypes are vectors in {{1, 1}, {1, 2}, {2, 2}}^ℓ^.  We thus use terms haplotype and genotype to refer to data over the whole marker map, and not e.g. to just one marker.

The allele of haplotype *H *at marker *i *is denoted by *H*(*i*). Similarly, the unordered allele pair of a genotype *G *at marker *i *is denoted by *G*(*i*). Given a pair of haplotypes {*H*_1_, *H*_2_} and a genotype *G *such that *G*(*i*) = {*H*_1_(*i*), *H*_2_(*i*)} for all *i*, we say that {*H*_1_, *H*_2_} is *compatible *with *G*, or that {*H*_1_, *H*_2_} is a (possible) *haplotype configuration *for genotype *G*. Two haplotypes determine a unique compatible genotype in the obvious way. A genotype, on the other hand, can have several compatible haplotype configurations. For a genotype *G *with *k heterozygous *markers (i.e., *k *= |{*G*(*i*) = {*a*_1_, *a*_2_} | *a*_1 _≠ *a*_2_}|; a marker is *homozygous *if it is not heterozygous), there are 2^*k *- 1 ^different haplotype configurations. The set of all haplotype configurations for a genotype *G *is denoted by *C*_*G*_, with |*C*_*G*_| = 2^*k *- 1^. A single haplotype *H *is said to be compatible with a genotype *G*, if there exists a haplotype H' such that {H, H'} is compatible with *G*. The set of input genotypes is denoted by G
 MathType@MTEF@5@5@+=feaafiart1ev1aaatCvAUfKttLearuWrP9MDH5MBPbIqV92AaeXatLxBI9gBamrtHrhAL1wy0L2yHvtyaeHbnfgDOvwBHrxAJfwnaebbnrfifHhDYfgasaacH8akY=wiFfYdH8Gipec8Eeeu0xXdbba9frFj0=OqFfea0dXdd9vqai=hGuQ8kuc9pgc9s8qqaq=dirpe0xb9q8qiLsFr0=vr0=vr0dc8meaabaqaciaacaGaaeqabaWaaeGaeaaakeaaimaacqWFge=raaa@382D@. The haplotype reconstruction problem is now defined as finding for each genotype *G *∈ G
 MathType@MTEF@5@5@+=feaafiart1ev1aaatCvAUfKttLearuWrP9MDH5MBPbIqV92AaeXatLxBI9gBamrtHrhAL1wy0L2yHvtyaeHbnfgDOvwBHrxAJfwnaebbnrfifHhDYfgasaacH8akY=wiFfYdH8Gipec8Eeeu0xXdbba9frFj0=OqFfea0dXdd9vqai=hGuQ8kuc9pgc9s8qqaq=dirpe0xb9q8qiLsFr0=vr0=vr0dc8meaabaqaciaacaGaaeqabaWaaeGaeaaakeaaimaacqWFge=raaa@382D@ the most plausible haplotype configuration {*H*_1_, *H*_2_} ∈ *C*_*G*_. Here, the interpretation of "most plausible" depends on each different haplotyping method; in most methods the most plausible haplotype configuration is the one with the highest estimated probability under the prior assumptions made by the method. In the case that multiple haplotype configurations are equally probable, we simply select one randomly.

## Results

### Haplotype probability models

Our focus is on data sets with a large number of relatively sparsely spaced markers. Under these conditions, recombinations between markers are common, and linkage disequilibrium between distant markers is weak. In this case it cannot be expected that complete haplotypes are shared between subjects; instead we aim to discover and utilize local regularities, or *patterns *in the haplotypes. We restrict our attention to a simple class of patterns: a *haplotype fragment *is a haplotype restricted to a (continuous) sub-range of the original marker map. The idea is to model local linkage disequilibrium by estimating the frequencies of haplotype fragments, and to combine those frequencies into a probability model for complete haplotypes. We here very briefly describe three different haplotype probability models based on this idea; full specifications are given in the Methods section. We introduced the ideas for two of them (the Markovian models) in a preliminary conference paper [22], one (the segmentation model) is completely novel. The actual haplotyping algorithm has also been greatly improved, resulting in significantly more accurate results and reduced running times, while scaling to much larger data sets. An overview of the algorithm is given in the Methods section; a detailed description with pseudo-code and complexity analysis is given in Additional file 1.

Let *H*(*i*, *j*) denote the sequence, or haplotype fragment, from the *i*th to the *j*th marker in a given haplotype *H*. In the *variable-order Markov model *the conditional probabilities at each marker *i *are estimated from fragments *H*(*s*_*i*_, i - 1) of varying length:

P(H)=P(H(1))∏i=2,...,ℓP(H(i)|H(si,i−1)).     (1)
 MathType@MTEF@5@5@+=feaafiart1ev1aaatCvAUfKttLearuWrP9MDH5MBPbIqV92AaeXatLxBI9gBaebbnrfifHhDYfgasaacH8akY=wiFfYdH8Gipec8Eeeu0xXdbba9frFj0=OqFfea0dXdd9vqai=hGuQ8kuc9pgc9s8qqaq=dirpe0xb9q8qiLsFr0=vr0=vr0dc8meaabaqaciaacaGaaeqabaqabeGadaaakeaacqWGqbaucqGGOaakcqWGibascqGGPaqkcqGH9aqpcqWGqbaucqGGOaakcqWGibascqGGOaakcqaIXaqmcqGGPaqkcqGGPaqkdaqeqbqaaiabdcfaqjabcIcaOiabdIeaijabcIcaOiabdMgaPjabcMcaPiabcYha8jabdIeaijabcIcaOiabdohaZnaaBaaaleaacqWGPbqAaeqaaOGaeiilaWIaemyAaKMaeyOeI0IaeGymaeJaeiykaKIaeiykaKcaleaacqWGPbqAcqGH9aqpcqaIYaGmcqGGSaalcqGGUaGlcqGGUaGlcqGGUaGlcqGGSaalcqWItecBaeqaniabg+GivdGccqGGUaGlcaWLjaGaaCzcamaabmaabaGaeGymaedacaGLOaGaayzkaaaaaa@5A4D@

The length of the context *H*(*s*_*i*_, *i *- 1), and thus the order of the Markov chain, is individually adjusted for each position and each haplotype by choosing the longest matching context that has a predetermined minimum frequency.

The *segmentation model *considers each haplotype as a sequence of independent, non-overlapping fragments, and defines the probability of a haplotype to be the product of fragment probabilities. A robust estimate is obtained by averaging over the set S
 MathType@MTEF@5@5@+=feaafiart1ev1aaatCvAUfKttLearuWrP9MDH5MBPbIqV92AaeXatLxBI9gBamrtHrhAL1wy0L2yHvtyaeHbnfgDOvwBHrxAJfwnaebbnrfifHhDYfgasaacH8akY=wiFfYdH8Gipec8Eeeu0xXdbba9frFj0=OqFfea0dXdd9vqai=hGuQ8kuc9pgc9s8qqaq=dirpe0xb9q8qiLsFr0=vr0=vr0dc8meaabaqaciaacaGaaeqabaWaaeGaeaaakeaaimaacqWFse=uaaa@3845@ of all possible segmentations of *H *into frequent fragments:

P(H)=C−1∑S∈Sq|S|−1⋅∏(si,ei)∈SP(H(si,ei)),     (2)
 MathType@MTEF@5@5@+=feaafiart1ev1aaatCvAUfKttLearuWrP9MDH5MBPbIqV92AaeXatLxBI9gBamrtHrhAL1wy0L2yHvtyaeHbnfgDOvwBHrxAJfwnaebbnrfifHhDYfgasaacH8akY=wiFfYdH8Gipec8Eeeu0xXdbba9frFj0=OqFfea0dXdd9vqai=hGuQ8kuc9pgc9s8qqaq=dirpe0xb9q8qiLsFr0=vr0=vr0dc8meaabaqaciaacaGaaeqabaWaaeGaeaaakeaacqWGqbaucqGGOaakcqWGibascqGGPaqkcqGH9aqpcqWGdbWqdaahaaWcbeqaaiabgkHiTiabigdaXaaakmaaqafabaGaemyCae3aaWbaaSqabeaacqGG8baFcqWGtbWucqGG8baFcqGHsislcqaIXaqmaaaabaGaem4uamLaeyicI4mcdaGae8NeXpfabeqdcqGHris5aOGaeyyXIC9aaebuaeaacqWGqbaucqGGOaakcqWGibascqGGOaakcqWGZbWCdaWgaaWcbaGaemyAaKgabeaakiabcYcaSiabdwgaLnaaBaaaleaacqWGPbqAaeqaaOGaeiykaKIaeiykaKcaleaacqGGOaakcqWGZbWCdaWgaaadbaGaemyAaKgabeaaliabcYcaSiabdwgaLnaaBaaameaacqWGPbqAaeqaaSGaeiykaKIaeyicI4Saem4uamfabeqdcqGHpis1aOGaeiilaWIaaCzcaiaaxMaadaqadaqaaiabikdaYaGaayjkaiaawMcaaaaa@6D77@

where *S *is a segmentation of *H *into (non-overlapping) segments (*s*_*i*_, *e*_*i*_), *q *is a parameter for penalizing large numbers of segments, |*S*| is the number of segments in segmentation *S*, and *C *is a normalization factor,

As a simpler alternative, we also consider *d-order Markov models *with

P(H)=P(H(1,d))∏i=d+1,...,ℓP(H(i)|H(i−d,i−1)).     (3)
 MathType@MTEF@5@5@+=feaafiart1ev1aaatCvAUfKttLearuWrP9MDH5MBPbIqV92AaeXatLxBI9gBaebbnrfifHhDYfgasaacH8akY=wiFfYdH8Gipec8Eeeu0xXdbba9frFj0=OqFfea0dXdd9vqai=hGuQ8kuc9pgc9s8qqaq=dirpe0xb9q8qiLsFr0=vr0=vr0dc8meaabaqaciaacaGaaeqabaqabeGadaaakeaacqWGqbaucqGGOaakcqWGibascqGGPaqkcqGH9aqpcqWGqbaucqGGOaakcqWGibascqGGOaakcqaIXaqmcqGGSaalcqWGKbazcqGGPaqkcqGGPaqkdaqeqbqaaiabdcfaqjabcIcaOiabdIeaijabcIcaOiabdMgaPjabcMcaPiabcYha8jabdIeaijabcIcaOiabdMgaPjabgkHiTiabdsgaKjabcYcaSiabdMgaPjabgkHiTiabigdaXiabcMcaPiabcMcaPaWcbaGaemyAaKMaeyypa0JaemizaqMaey4kaSIaeGymaeJaeiilaWIaeiOla4IaeiOla4IaeiOla4IaeiilaWIaeS4eHWgabeqdcqGHpis1aOGaeiOla4IaaCzcaiaaxMaadaqadaqaaiabiodaZaGaayjkaiaawMcaaaaa@5F4C@

More details on each of these models are given in the Methods section.

### Experimental setting

#### Data simulation

Our main target application is data sets involving a large number of subjects (hundreds or thousands) and markers (hundreds or thousands per chromosome), because these are needed for association-based gene mapping [18]. Such data is not yet publicly available for benchmarking, and thus our experiments are based mainly on simulated data. Real data from the HapMap project [23] is used to validate the general observations from simulations, and to test the method in slightly different settings, especially with small sample sizes.

We used Hudson's coalescence simulator [24] to simulate data sets of 1000 genotypes. Our simulated settings range from 5 to 500 markers, with average marker spacings between 6.6 and 166 kb and map lengths between 166 kb and 16.6 Mb. These marker distances correspond to genome-wide studies with 500 k to 20 k markers; the average linkage disequilibrium between neighboring markers, measured with Lewontins |*D'*| measure, ranges respectively from 0.88 to 0.36 (Table 1). More details on the data simulation procedure are given in the Methods section.

**Table 1:** Correspondence between marker spacing and linkage disequilibrium.

Marker spacing (kb)	|*D'*|	number of markers
6.6 kb	0.88	500 k
20 kb	0.73	166 k
33 kb	0.64	100 k
100 kb	0.45	33 k
166 kb	0.36	20 k

Average marker distance, average linkage disequilibrium (|D'|) between adjacent markers, and the corresponding number of markers in a genome-wide study; simulated data.

All experiments are run separately for 10 independently simulated data sets, and we report average results over them. Results from the different replicates are quite similar: while there is some variance in the accuracy between the different replicas, the relative performances of different methods are extremely similar. (See Additional file 2 for example results from all 10 replicas.) Unless otherwise stated, 100 markers were used in the experiments with HaploRec alone, and 30 markers in comparisons to other methods, in order to keep running times of some of the other methods reasonable.

#### Performance measure

As an accuracy measure, we primarily use relative switch accuracy, which is defined as the fraction of neighboring phases (between each pair of consecutive heterozygous markers) reconstructed correctly. An alternative measure would be absolute accuracy, which is the fraction of haplotype pairs reconstructed completely correctly (ignoring missing alleles). Absolute accuracy is problematic with long or sparse haplotypes, where some switch errors can be inevitable. It gives little information about the quality of a haplotype, except whether it is exactly correct or not; the switch measure is much more informative in this respect. Chromosome-wide studies search for relatively short disease-associated haplotype fragments, and switch accuracy is almost directly related to the number of fragments correctly reconstructed. However, we also give some examples of absolute errors since they are widely used in the field (for short haplotypes).

### Experimental results

#### Performance across different amounts of linkage disequilibrium

The three proposed haplotype probability models, Markov chain of variable order (VMM), the segmentation model (S), and as simpler baseline the Markov chain of fixed order (FMM), lead to different variants of HaploRec, subsequently abbreviated as HaploRec-VMM, HaploRec-S, and HaploRec-FMM. The most important parameter of HaploRec is the one that indirectly specifies the size of the data structure used by the model (subsequently called *model complexity parameter*). For the variable-order Markov model and the segmentation model this parameter is the fragment frequency threshold, with the fixed-order Markov model, it is the order of the Markov model (for details, see the Methods section).

**Figure 1:**
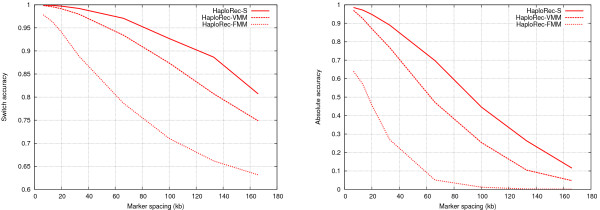
**Comparison of FMM, VMM and segmentation models**. Switch accuracy on the left, absolute accuracy on the right.

The optimal value of the model complexity parameter depends on the amount of linkage disequilibrium, so a fixed value does not give comparable results across different marker spacings. In the first experiment, we compare the proposed models to each other, by testing each model for a large range of different values (minimum frequency 1–15 for the variable-order Markov and segmentation models, order 1–17 for the fixed-order Markov model). The optimal value was then chosen for each marker spacing separately. In the experiment, the accuracy of the models under different amounts of linkage disequilibrium was tested using 100 markers with average spacing between 6.6 and 166 kb (Figure 1)

The segmentation model performs here best, variable-order Markov model is slightly less accurate, and the fixed-order model is clearly inferior. This and other experiments (not shown) show that the more complex models are good alternatives over the simple Markov model. For clarity of exposition, the fixed-order Markov model is excluded from the rest of the results as markedly inferior. We also considered a variant of the segmentation model where the maximum over different segmentations was used, instead of averaging over different segmentations. This alternative was slightly but consistently outperformed by the averaging model (results not shown).

#### Default values for model complexity parameters

**Figure 2:**
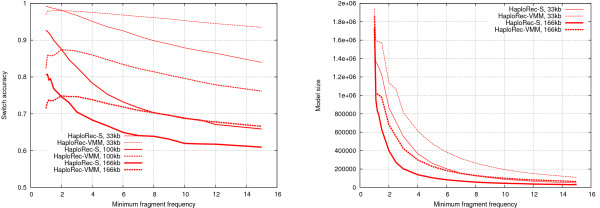
**Effect of minimum frequency on the VMM and segmentation models**. Switch accuracy on the left, model size on the right. (We use red color throughout this paper for HaploRec; solid line for the segmentation model and dashed line for the variable order model.)

To make later comparisons to other methods fair, we must fix the minimum frequency parameter to a fixed default value for each of the models. Consider now the accuracies and model sizes (number of stored haplotype fragments) as functions of the minimum frequency threshold, for three different marker spacings: 33, 100 and 166 kb (Figure 2). With the segmentation model, decreasing the threshold always improves results. A threshold of 1.2 was chosen for the subsequent experiments as a compromise between efficiency and accuracy (note that the frequency threshold does not need to be an integer, as frequencies of fragments are estimated from reconstructed haplotypes which may have non-integer probabilities; see Methods for more details). With the variable-order Markov model the optimal choice seems to depend more on the amount of linkage disequilibrium. For the Markov model, a threshold of 2 was chosen for the rest of the experiments.

#### Comparison of HaploRec with fastPhase, Gerbil, Phase, PL-EM, and Snphap

**Figure 3:**
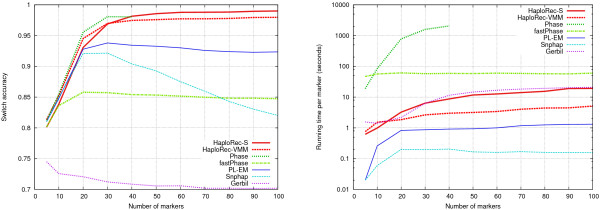
**Comparison of methods, variable number of markers**. Switch accuracy on the left, running times (plotted with logarithmic scale) on the right.

For the rest of this section we study how HaploRec performs in comparison to existing statistical haplotyping methods. Five publicly available haplotyping programs were chosen for benchmarking: fastPhase [21] (version 1.1.3), Gerbil [10] (version 1.0), Phase [7,8] (version 2.0), PL-EM [6] (version 1.5, kindly provided by Zhaohui S. Qin) and Snphap [19] (version 1.3.1). The current version of HaploRec, 2.1, was used in the experiments. FastPhase, Gerbil and Snphap were run with their default parameter values. For PL-EM, buffer size was set to 300, number of iterations to 20, and parsize to 2. For Phase, the number of iterations and burn-in iterations were both set to 100, the thinning interval was set to 1, and the recombination model introduced in version 2.0 was used. All experiments were run on PCs with a 2.8 GHz Pentium 4 processor and 1 GB of memory. All methods were tested on a range of data sets containing 5 to 100 markers with 33 kb spacing (Figure 3; Table 2). HaploRec-S reaches the best accuracy, 99.0% (with windows of 100 markers), closely followed by Phase (98.1% accuracy with windows of 30 markers) and HaploRec-VMM (98.0% accuracy with windows of 100 markers); the HaploRec variants are several orders of magnitude faster. PL-EM, Snphap, and Gerbil are clearly less accurate but faster; fastPhase is both less accurate and slower than HaploRec. Phase aborted because of running out of memory (1 GB) when there were more than 40 markers.

**Table 2:** Comparison of methods on simulated data sets with 33 kb marker spacing.

Method	Switch accuracy	No. of markers	Time per marker (sec.)
HaploRec-S	0.990	100	19.2
HaploRec-VMM	0.980	100	5.2
(HaploRec-S)	(0.969)	(30)	(6.6)
(HaploRec-VMM)	(0.970)	(30)	(2.5)
Phase	0.981	30	1585
fastPhase	0.858	20	61.3
PL-EM	0.938	30	0.9
Snphap	0.921	30	0.4
Gerbil	0.745	5	2.6

For each method, the table gives the accuracy, the number of markers to be haplotyped at a time to achieve that accuracy, and the running time per marker. As 30 marker windows are optimal for many methods, results for them are given in parenthesis also for the HaploRec variants.

An observation from this experiment is that the number of markers can have a significant effect on haplotyping accuracy. With the smallest tested numbers of markers, 5 and 10, all the methods achieve only a mediocre accuracy. When the number of markers is increased to 20 or 30, the accuracies of all methods except Gerbil improve clearly. The accuracy of both HaploRec variants increases monotonically with the number of markers. None of the other methods show this property. Phase's accuracy decreases slightly after 30 markers (and it was not able to handle more than 40 markers). Snphap and PL-EM gain less from the increase in the number of markers, and also start to actually lose accuracy when the number of markers increases over 30. FastPhase only gains little and levels after 20 markers.

Snphap, the HaploRec variants, and Gerbil are relatively close to having linear running times in the number of markers (i.e., close to a horizontal line on the scale where *y*-axis gives the time per marker; Figure 3, right panel). Snphap is 1–2 orders of magnitude faster than PL-EM, HaploRec and Gerbil; they are an order of magnitude faster than fastPhase, which in turn is 0–2 orders of magnitude faster than Phase. For clarity of exposition and fairness of comparison, we use 30 marker windows in the rest of the experiments for all methods. Based on the results above, it is about an optimal choice for Phase, fastPhase, PL-EM, and Snphap, slightly suboptimal for HaploRec, and unfortunately bad for Gerbil. In particular, note that if a larger window size was chosen for HaploRec, HaploRec-S could in many cases have given more accurate results than Phase (cf. Table 2).

#### Effect of marker density

**Figure 4:**
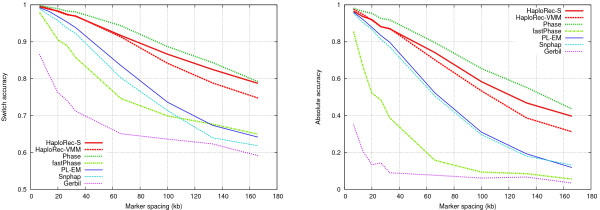
**Comparison of methods, variable marker density**. Switch accuracy on the left, absolute accuracy on the right.

We next evaluated the performance of the methods as a function of the amount of linkage disequilibrium, resulting from varying marker spacing between 6.6 and 166 kb (Figure 4, cf. Table 1). The accuracy of all methods decreases with increasing marker spacing, as expected. Phase and the two HaploRec variants are clearly more resistant to decreasing linkage disequilibrium than the other methods. FastPhase and Gerbil have a drop in accuracy at first, but then come close to PL-EM and Snphap with the sparsest maps. Marker spacing also has a clear effect on running times for some of the methods (not shown): for Phase, PL-EM and Gerbil, the difference of running times between the densest (6.6 kb) and sparsest (133 kb) settings is approximately 40-fold. For HaploRec and Snphap, running times only vary by a factor of 1.5 – 3 (in the case of HaploRec, this is explained by the increased number of iterations needed for convergence), while fastPhase's running times are practically same for all spacings.

#### Effect of sample size

**Figure 5:**
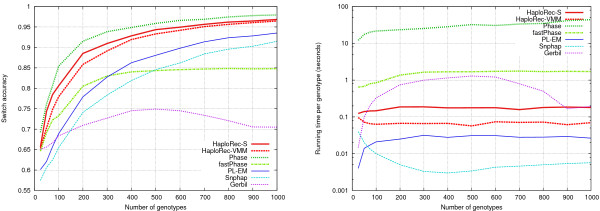
**Effect of sample size**. Switch accuracy on the left, running times (on a logarithmic scale) on the right.

The available sample size obviously has an effect on the difficulty of the task. Most of the evaluations of haplotyping methods in the literature have been made on very small data sets, and thus it can be interesting to see how sample size effects different methods. Tests with sample sizes between 25 and 1000 individuals clearly show that all methods perform better with larger sample sizes, but there are marked differences between methods (Figure 5; 30 markers with 33 kb spacing). Phase, HaploRec, PL-EM and Snphap are able to benefit from larger sample sizes. FastPhase and Gerbil gain much less from larger samples: while they perform approximately equally to HaploRec with very small sample sizes, HaploRec is more accurate by a large margin with larger sample sizes. Also, Phase seems to work relatively well also with the smallest sample sizes.

For most methods, the running times are relatively close to linear in the number of genotypes, but Phase's running times increase somewhat more rapidly. We performed some additional experiments with fastPhase, trying out different parameter values (not shown). In particular, increasing the maximum number of clusters from the default value of 15 to 20 or 30 improved the accuracy at the cost of longer running times, but the accuracies did not reach those of HaploRec.

#### Effect of missing alleles and genotyping errors

**Figure 6:**
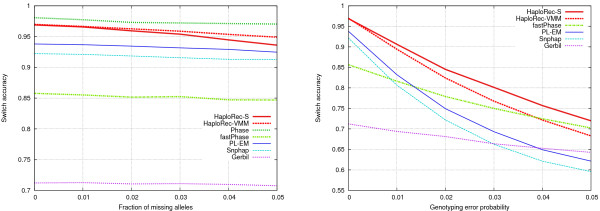
Effect of missing alleles (left) and genotyping errors (right).

Two sets of experiments were performed to see how up to 5% missing alleles or up to 5% genotyping errors affect the accuracy. According to our experience, such numbers are typical for current high-quality genotypes. The number of markers was again fixed to 30 and marker spacing was 33 kb (Figure 6). Phase failed to complete when the error fraction was more than 2%, due to increased memory usage resulting from the increased haplotype diversity. Also, the running time of Phase increased rapidly with increasing error fraction; already with 2%, Phase ran for approximately 4 days for a single replica. For this reason, results for Phase are not shown in the error fraction figure. Overall, missing alleles have only a slightly negative effect on accuracy of all tested methods. HaploRec-S seems to be slightly more sensitive to missing data than the other methods. Genotyping errors, on the other hand, have a more clear effect on accuracy. HaploRec-VMM, PL-EM and Snphap experience a clear drop in accuracy, whereas HaploRec-S, fastPhase and Gerbil are more robust against genotyping errors. In this setting, the results start to be unacceptable between about 1% (Snphap, PL-EM, fastPhase) and 2% (HaploRec) of errors.

#### Tests with larger data sets

**Figure 7:**
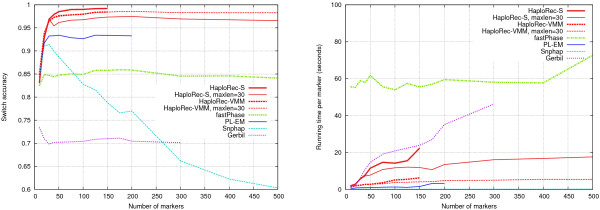
**Accuracies and running times for large data sets**. Switch accuracy on the left, running times on the right.

The applicability of the methods for larger amounts of markers and longer genomic regions was tested by varying the number of markers from 10 to 500 (with the map growing at the same time from 330 kb to 16.5 Mb, with a fixed marker spacing of 33 kb; Figure 7). When the length of haplotype fragments was not limited, HaploRec reaches the 1 GB memory limit at about 150 markers. To work with a larger number of markers, HaploRec was also run with the maximum fragment length set to 30, which significantly reduced memory usage, enabling it to handle all tested window sizes. Also some of the other methods had a practical maximum number of markers they could handle on these data sets; 200 for PL-EM and 300 for Gerbil. Phase was excluded from this experiment, as it was already observed that it was unable to handle data sets with more than 40 markers.

HaploRec-S without the maximum pattern length constraint has the best accuracy, but its high memory usage makes it unusable for the larger window sizes. On the other hand, the segmentation model does not work quite as well with the pattern length constraint. HaploRec-VMM has a fairly good accuracy, and is almost unaffected by the pattern length constraint (the lines for the two variants are indistinguishable in the figure), making it practical for large data sets. Its accuracy stays approximately constant with 100 markers or more. FastPhase and PL-EM maintain a lower but constant accuracy. Gerbil works up to 300 markers and maintains its (low) level of accuracy. While Snphap quickly loses accuracy with an increasing number of markers, it is very fast (its running times per marker are below a second). The running times of the HaploRec variants and PL-EM are slightly superlinear (i.e., approaching a horizontal line; note that the *y*-axis shows the time per marker) and Gerbil clearly superlinear.

#### Experimental results with real data

To complement the systematic experimental analysis with several replicates of simulated data and reasonably sized samples, the methods were also tested on publicly available real data from the HapMap project [23].

The HapMap data we used consists of two separate populations: 30 trios from the Yoruba population in Ibadan, Nigeria, and another 30 trios from the CEPH population (Utah residents with ancestry from northern and western Europe). Both data sets (downloaded from the HapMap web site [25]) have the same set of 3.8 million SNPs spread over the whole genome. (For information on how HapMap data was processed for the experiments, see the Methods section.)

In the following experiments with this data, HaploRec was run with the same parameter values as before, chosen based on the first experiments with large samples of simulated data (Figures 1 and 2).

**Figure 8:**
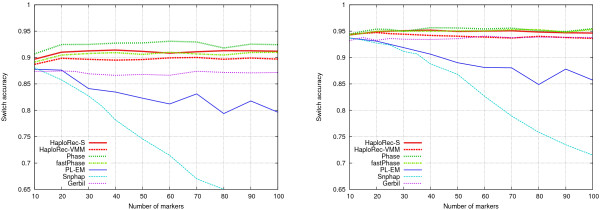
**Accuracies on HapMap data, variable number of markers**. Marker spacing 6.0 kb. Yoruba on the left, CEPH on the right.

In the first experiment, the marker spacing was fixed to 6 kb (which corresponds roughly to a genome-wide study with 500 k markers). The number of markers was varied from 10 to 100 to test the relationship between the number of markers and haplotyping accuracy (Figure 8, Yoruba data on the left, CEPH data on the right).

The younger CEPH population is clearly easier than the Yoruba population for all haplotyping methods. However, for both data sets, even the best methods only achieve a relatively low accuracy, which can probably be attributed to the small sample size. For the Yoruba data, Phase performs best across the range of tests, followed by the HaploRec variants and fastPhase, which are followed by Gerbil, PL-EM and Snphap. For the CEPH data, the differences between the best methods (Phase, HaploRec-S and fastPhase) are very small.

The above results show that HaploRec is competitive also on real data sets having a small sample size. On the other hand, the number of marker has a smaller effect on accuracy than in the simulated data sets. Also, increasing the number of markers to more than 40 does not improve accuracy here, unlike in the experiments with simulated data sets, where accuracy always improves with increasing number of markers. We believe the reason for this is the small sample size (60 genotypes vs. 1000 genotypes in the simulated data). In a larger sample, longer shared ancestral haplotype fragments (which are rarer than shorter ones) can be detected and utilized, leading to increased accuracy. To test this hypothesis, we ran HaploRec on (sparse) simulated data sets consisting of various numbers of genotypes and evaluated the effect of the number of markers (data not shown). The same effect was visible there as well: the improvement in accuracy gained by using more markers decreases with decreasing sample size.

**Figure 9:**
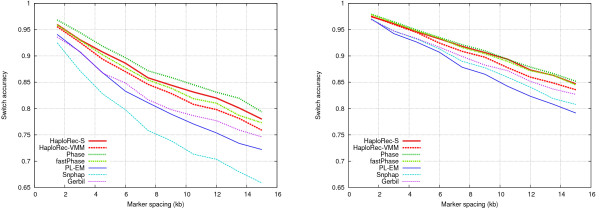
**Accuracies on HapMap data, variable marker spacing**. 30 markers. Yoruba on the left, CEPH on the right.

In a second experiment with the HapMap data, we varied marker spacing between 1.5 and 15 kb, while the number of markers was fixed to 30 (Figure 9, Yoruba data on the left, CEPH data on the right). The accuracy drops with increasing marker spacing, as expected, but the methods roughly maintain their relative performances, in the order Phase, HaploRec-S, fastPhase, HaploRec-VMM, Gerbil, PL-EM and Snphap. Again, for the CEPH data, the best three methods have practically identical accuracies.

## Discussion

The HaploRec models and algorithm introduced in this paper are designed for haplotyping data sets in large-scale disease association studies. The statistical models are relatively simple. Their central idea is to represent the probability of a long haplotype as a function of the probabilities of its local fragments. Our experimental results confirm that locality and simplicity were successful design choices: locality allows the models to actually benefit from large numbers of markers even when they are sparsely located, while simplicity allows an efficient implementation. In combination these properties result in a method that accurately and quickly haplotypes large windows of markers at a time.

We presented three different haplotype probability models: Markov chain of a fixed order, Markov chain of a variable order (HaploRec-VMM), and a model based on segmenting each haplotype in frequent fragments (HaploRec-S). Our experimental results show that the simple fixed-order Markov chain is clearly inferior, even if its order is chosen optimally for the data set. In our results for the more flexible and "self-adjusting" variable-order and segmentation models, the segmentation model gave generally more accurate results than the variable-order Markov model both in the simulated and real data sets, with the downside of being somewhat slower. Our experiments also indicated another interesting difference between the models: while the segmentation model is more sensitive to missing data than the variable-order Markov model, it is more robust against genotyping errors.

In experimental comparisons with existing haplotyping methods, the HaploRec models scale in a unique way to large data sets: Their accuracies improve both in the number of markers (Figure 3) and in the sample size (Figure 5) in an unparalleled way (Phase being an exception in some aspects), while being robust to sparse marker maps (Figure 4). This combination of properties makes HaploRec especially suitable for chromosome or genome-wide association analysis, where large numbers of sparsely located markers are analyzed for hundreds or thousands of individuals. In such settings, HaploRec can outperform Phase in accuracy while being 2–3 orders of magnitude faster. Although Phase is very accurate and can also benefit from large samples, it is computationally very intensive, restricting its usability for large data sets. The recently proposed fastPhase method scales well but for large data sets, it seems to be clearly less accurate and also clearly slower than HaploRec.

The performance differences can be largely understood from the properties of the different models. Snphap and PL-EM are based on a multinomial model, which is suitable when all markers are in strong linkage disequilibrium, but does not work well when the number of markers is large or the markers are spaced far apart. Gerbil solves this problem by dividing the marker map into blocks and modeling each block by a set of common "founder" haplotypes. This approach assumes strong linkage disequilibrium within each block, and does not account for more flexible patterns of linkage disequilibrium across block boundaries. Gerbil's relatively bad performance on simulated data can probably be attributed to two factors. Firstly, it only considers a small number of common haplotypes for each block, and thus cannot utilize the rarer shared haplotypes found in large samples. Secondly, there are no recombination hot-spots in the simulated data. In fastPhase, each haplotype is a mosaic of founder haplotypes. This allows for both block-like patterns of linkage disequilibrium and gradual decay of linkage disequilibrium with distance. However, it does not account for the fact that shared haplotypes are formed also in the later stages of the population history, forming more recent (and less frequent) patterns of linkage disequilibrium. The segmentation model of HaploRec bears some resemblance to the model used in fastPhase: each haplotype is a mosaic of frequent haplotype fragments, which may now be ancestral haplotype fragments from different stages of the population history. As old haplotypes are split by recombinations and new haplotypes are formed during the population history, longer shared fragments probably are of more recent origin and have a smaller frequency than shorter ones. HaploRec is not constrained to a fixed set of founders, but can more flexibly utilize (local) patterns of linkage disequilibrium present in the data using fragments of varying lengths. Being able to utilize the information contained in rare shared fragments is one possible explanation for the good performance of HaploRec on large samples. Phase works consistently well across most settings. Also its model accounts for recombinations, which explains why it performs well also on sparser marker maps. Its good accuracy especially on small samples is probably due to its more realistic prior compared to other methods, which becomes less important with increasing sample size. The accurate model employed by Phase has the downside of being computationally very intensive. As a result, the window size (number markers) it can handle is very limited, especially when the sample size is large. In the experiments with variable window size, we also observed that Phase's accuracy decreases when increasing the number of markers above 30; this may be because its default number of iterations is not sufficient to obtain mixing for larger window sizes.

Results with data from the HapMap project demonstrate that HaploRec works well also with real data (Figures 8 and 9). Unlike the assumed primary applications of HaploRec, these samples are very small (only 60 genotypes). Still, HaploRec is very competitive, even with its default parameter values chosen based on the simulated, larger data sets with sparser marker spacing. Experiments with the dense Daly data [26] (results not shown) indicate the same: among the tested methods, HaploRec was second only to Phase and fastPhase while being fastest of all methods. Future experiments with large real data sets, as they become publicly available, will be used to test the hypothesis that HaploRec will benefit more than the competing methods from larger sample sizes and from more markers.

For evaluating performance on reasonably large samples (1000 individuals), we used simulated data due to the lack of suitable public real data sets. Another option would have been to use pseudo-simulated data, by randomly combining pairs of real haplotypes to form new pseudo-individuals (as done, e.g., in [10]). For the genetically long marker maps of association studies, this method would generate unrealistic data: multiple copies of complete haplotypes will appear in the final sample, when in reality the haplotypes would mostly have been segmented by recombinations. Such data would be unrealistically easy to haplotype, and already a simple multinomial model can give almost completely correct solutions.

Most haplotype-based association analysis methods assume haplotypes as input. There are several major fundamental issues related to this. The first questions the need to haplotype longer windows of markers maps at all, since gene mapping studies search for disease associations of relatively short genetic regions and for this it is sufficient to haplotype such a shorter segment at a time. The computational complexity can on one hand decrease – especially for methods that are not close to linear in the number of markers, such as Phase – but on the other hand there is an additional cost of redundantly haplotyping overlapping segments. Additionally, haplotyping accuracy improves with window size (cf. Figures 3 and 8), indicating that too short windows should be avoided.

The second issue relates to the use of estimated haplotypes in association analysis. It seems obvious that haplotype reconstruction tends to exaggerate linkage disequilibrium since haplotyping methods more or less directly aim to maximize it. It has been shown that estimated haplotypes can, indeed, lead to false positives [27,28]. On the other hand, this does not always have to be the case. A simulation study shows that in association analysis, the haplotypes produced by HaploRec can be equally powerful to the true haplotypes, despite some inevitable phasing errors [29]. The locality of the statistical models has a subtle role here: in the case-control settings normally used in association studies, linkage disequilibrium is increased in the cases in the vicinity of the disease gene, making this most critical part of the marker map easier to haplotype. More work is needed to identify when statistically predicted haplotypes are useful and when not.

Another view to this issue is that some information is lost. As an extreme example, there may be several roughly equally likely haplotype configurations of which just one is chosen to the output. Since association analysis methods are typically based on frequencies of haplotypes (or fragments), the frequencies of different possible haplotypes – rather than the single most likely ones – should be more informative and fairly easily usable for many association methods. Most statistical haplotyping methods internally estimate haplotype frequencies; haplotype resolution can be seen as an extra step based on these frequencies. This also holds for HaploRec which actually already estimates frequencies of all haplotype fragments that have (an estimated) frequency above a small threshold. Ultimately, haplotype frequency estimation and association analysis could be combined into one model and process [27].

There are several possible directions for future work on improving the haplotype probability models. Currently, the variable-order Markov model only uses a simple frequency threshold to determine the context lengths. The set of contexts could be pruned further using the accuracy of predicting the next allele as a selection criterion [30]. Another possibility for refinement is to smooth the probability over several context lengths simultaneously [31]. Ideas from the models of Phase and Gerbil could be used to better account for mutations and genotyping errors. A possible approach would be to allow for a small number of mismatches between the haplotype fragments and complete haplotypes in the parameter estimation step, dividing some of the probability mass to fragments that are similar, but not identical with the observed ones.

## Conclusion

Genotyping hundreds or even thousands of subjects for hundreds of thousands of markers is becoming technologically and economically feasible. It is estimated that data sets of this size start to be sufficiently powerful for genome-wide disease association studies, depending on the disease and the population [18,32]. However, many methods for association-based gene mapping assume haplotype data. It has been shown, too, that haplotypes can be more powerful than single markers [33].

We presented models and methods for statistical haplotype reconstruction from genotypes of unrelated individuals, and specifically targeted large and sparse data sets, such as those needed in chromosome or genome-wide disease association studies. We introduced three different haplotype probability models: Markov chain of a fixed order, Markov chain of a variable order, and a model based on segmenting each haplotype into frequent fragments. In Methods we give full specifications of the models and an concise description of the HaploRec algorithm; Additional file 1 contains a more detailed description of the algorithm and complexity analysis.

Experiments with simulated and real data demonstrate that these models and methods, collectively called HaploRec, are competitive with existing methods in terms of accuracy while being several orders of magnitude faster than the most accurate competitors. Of the two HaploRec models, the segmentation model is recommended as the default choice, as it generally gives more accurate results. However, for very large data sets, or when there is much missing data, the variable-order Markov model may be a better alternative, due to its smaller computational demands and smaller sensitivity to missing data.

## Methods

### Haplotype probability models

Recall that *H*(*i*, *j*) denotes the sequence (haplotype fragment) from the *i*th to the *j*th marker in a given haplotype *H*. We use the alternative notation *frag*(*h*, *i*, *j*) to denote a haplotype fragment from *i *to *j*, consisting of marker string *h*, when the fragment is not a projection from any particular haplotype *H*. We will denote *H*(*i*, *i*), a fragment consisting of a single marker, simply by *H*(*i*). Similarly, *G*(*i*, *j*) denotes the sequence of allele pairs from the *i*th to the *j*th marker in genotype *G*, called *genotype fragment*. Again, *G*(*i*, *i*) is denoted by *G*(*i*). We say that a fragment *H*(*i*, *j*) and a haplotype *H' match *if *H*(*k*) = *H'*(*k*) for all *k *: *i *≤ *k *≤ *j*. We say that a fragment *H*(*i*, *j*) and a genotype *G match *if there exists a string H¯
 MathType@MTEF@5@5@+=feaafiart1ev1aaatCvAUfKttLearuWrP9MDH5MBPbIqV92AaeXatLxBI9gBaebbnrfifHhDYfgasaacH8akY=wiFfYdH8Gipec8Eeeu0xXdbba9frFj0=OqFfea0dXdd9vqai=hGuQ8kuc9pgc9s8qqaq=dirpe0xb9q8qiLsFr0=vr0=vr0dc8meaabaqaciaacaGaaeqabaqabeGadaaakeaacuWGibasgaqeaaaa@2DDD@ ∈ Π_*k *= *i*,...,*j *_*A*_*k *_such that {*H*(*i*, *j*), H¯
 MathType@MTEF@5@5@+=feaafiart1ev1aaatCvAUfKttLearuWrP9MDH5MBPbIqV92AaeXatLxBI9gBaebbnrfifHhDYfgasaacH8akY=wiFfYdH8Gipec8Eeeu0xXdbba9frFj0=OqFfea0dXdd9vqai=hGuQ8kuc9pgc9s8qqaq=dirpe0xb9q8qiLsFr0=vr0=vr0dc8meaabaqaciaacaGaaeqabaqabeGadaaakeaacuWGibasgaqeaaaa@2DDD@} is compatible with *G*(*i*, *j*). Given a set of haplotypes, the frequency of a fragment *H*(*i*, *j*) is defined as the number of haplotypes matching the fragment, and is denoted by ℱ
 MathType@MTEF@5@5@+=feaafiart1ev1aaatCvAUfKttLearuWrP9MDH5MBPbIqV92AaeXatLxBI9gBamrtHrhAL1wy0L2yHvtyaeHbnfgDOvwBHrxAJfwnaebbnrfifHhDYfgasaacH8akY=wiFfYdH8Gipec8Eeeu0xXdbba9frFj0=OqFfea0dXdd9vqai=hGuQ8kuc9pgc9s8qqaq=dirpe0xb9q8qiLsFr0=vr0=vr0dc8meaabaqaciaacaGaaeqabaWaaeGaeaaakeaaimaacqWFXeIraaa@3787@(*H*(*i*, *j*)).

A simple model for haplotype probability is to consider the haplotype as a (first-order) Markov chain in which the probability for a marker having a certain allele depends only on the preceding marker:

P(H)=P(H(1))∏i=2,...,ℓP(H(i)|H(i−1)).     (4)
 MathType@MTEF@5@5@+=feaafiart1ev1aaatCvAUfKttLearuWrP9MDH5MBPbIqV92AaeXatLxBI9gBaebbnrfifHhDYfgasaacH8akY=wiFfYdH8Gipec8Eeeu0xXdbba9frFj0=OqFfea0dXdd9vqai=hGuQ8kuc9pgc9s8qqaq=dirpe0xb9q8qiLsFr0=vr0=vr0dc8meaabaqaciaacaGaaeqabaqabeGadaaakeaacqWGqbaucqGGOaakcqWGibascqGGPaqkcqGH9aqpcqWGqbaucqGGOaakcqWGibascqGGOaakcqaIXaqmcqGGPaqkcqGGPaqkdaqeqbqaaiabdcfaqjabcIcaOiabdIeaijabcIcaOiabdMgaPjabcMcaPiabcYha8jabdIeaijabcIcaOiabdMgaPjabgkHiTiabigdaXiabcMcaPiabcMcaPaWcbaGaemyAaKMaeyypa0JaeGOmaiJaeiilaWIaeiOla4IaeiOla4IaeiOla4IaeiilaWIaeS4eHWgabeqdcqGHpis1aOGaeiOla4IaaCzcaiaaxMaadaqadaqaaiabisda0aGaayjkaiaawMcaaaaa@5673@

The obvious shortcoming of this model is that although linkage disequilibrium is normally strongest between neighbors, it is not limited to the immediate neighboring markers; a neighborhood of several markers is thus potentially more informative. More power in predicting the next allele can thus be obtained by increasing the order *d *of the Markov model:

P(H)=P(H(1,d))∏i=d+1,...,ℓP(H(i)|H(i−d,i−1)).     (5)
 MathType@MTEF@5@5@+=feaafiart1ev1aaatCvAUfKttLearuWrP9MDH5MBPbIqV92AaeXatLxBI9gBaebbnrfifHhDYfgasaacH8akY=wiFfYdH8Gipec8Eeeu0xXdbba9frFj0=OqFfea0dXdd9vqai=hGuQ8kuc9pgc9s8qqaq=dirpe0xb9q8qiLsFr0=vr0=vr0dc8meaabaqaciaacaGaaeqabaqabeGadaaakeaacqWGqbaucqGGOaakcqWGibascqGGPaqkcqGH9aqpcqWGqbaucqGGOaakcqWGibascqGGOaakcqaIXaqmcqGGSaalcqWGKbazcqGGPaqkcqGGPaqkdaqeqbqaaiabdcfaqjabcIcaOiabdIeaijabcIcaOiabdMgaPjabcMcaPiabcYha8jabdIeaijabcIcaOiabdMgaPjabgkHiTiabdsgaKjabcYcaSiabdMgaPjabgkHiTiabigdaXiabcMcaPiabcMcaPaWcbaGaemyAaKMaeyypa0JaemizaqMaey4kaSIaeGymaeJaeiilaWIaeiOla4IaeiOla4IaeiOla4IaeiilaWIaeS4eHWgabeqdcqGHpis1aOGaeiOla4IaaCzcaiaaxMaadaqadaqaaiabiwda1aGaayjkaiaawMcaaaaa@5F50@

With *d *= 1 we obviously have the standard Markov chain as a special case. Selecting a suitable value for *d *can be a problem. Increasing the order increases accuracy of predicting the next allele, but only to a certain extent; at some point, the conditional probability can no longer be reliably estimated from a limited sample of haplotypes. Another problem with fixing *d *is the fact that linkage disequilibrium may vary within the marker map; it is thus possible that no single value of *d *is suitable for all parts of the map. A more flexible alternative to the fixed-order Markov model is to use a variable-order Markov model, where the context is adjusted for each marker and haplotype individually. Informally, the goal is to find a flexible balance between generality and informativeness. We propose the following solution. When we estimate the probability of a haplotype *H *and consider the variable-order Markovian distribution at marker *i*, we find the longest observed fragment that (1) matches haplotype *H *and ends at marker *i *- 1, and (2) has a frequency exceeding some given threshold, *minfr*. The fragments whose frequency does not exceed this threshold are considered uninformative. Using a frequency threshold is motivated by the fact that it is not likely that a long fragment of haplotypes is shared by different individuals unless it is inherited from the same ancestor; thus using only frequent fragments gives increased confidence in the fragments being identical by descent.

Given a frequency threshold *minfr*, we first compute the set of most frequent haplotype fragments, denoted by ℱ
 MathType@MTEF@5@5@+=feaafiart1ev1aaatCvAUfKttLearuWrP9MDH5MBPbIqV92AaeXatLxBI9gBamrtHrhAL1wy0L2yHvtyaeHbnfgDOvwBHrxAJfwnaebbnrfifHhDYfgasaacH8akY=wiFfYdH8Gipec8Eeeu0xXdbba9frFj0=OqFfea0dXdd9vqai=hGuQ8kuc9pgc9s8qqaq=dirpe0xb9q8qiLsFr0=vr0=vr0dc8meaabaqaciaacaGaaeqabaWaaeGaeaaakeaaimaacqWFXeIraaa@3787@_*minfr*_, which will determine the sizes of contexts:

ℱ
 MathType@MTEF@5@5@+=feaafiart1ev1aaatCvAUfKttLearuWrP9MDH5MBPbIqV92AaeXatLxBI9gBamrtHrhAL1wy0L2yHvtyaeHbnfgDOvwBHrxAJfwnaebbnrfifHhDYfgasaacH8akY=wiFfYdH8Gipec8Eeeu0xXdbba9frFj0=OqFfea0dXdd9vqai=hGuQ8kuc9pgc9s8qqaq=dirpe0xb9q8qiLsFr0=vr0=vr0dc8meaabaqaciaacaGaaeqabaWaaeGaeaaakeaaimaacqWFXeIraaa@3787@_*minfr *_= {*frag*(*h*, *i*, *j*) | 1 ≤ *i *≤ *j *≤ ℓ, ℱ
 MathType@MTEF@5@5@+=feaafiart1ev1aaatCvAUfKttLearuWrP9MDH5MBPbIqV92AaeXatLxBI9gBamrtHrhAL1wy0L2yHvtyaeHbnfgDOvwBHrxAJfwnaebbnrfifHhDYfgasaacH8akY=wiFfYdH8Gipec8Eeeu0xXdbba9frFj0=OqFfea0dXdd9vqai=hGuQ8kuc9pgc9s8qqaq=dirpe0xb9q8qiLsFr0=vr0=vr0dc8meaabaqaciaacaGaaeqabaWaaeGaeaaakeaaimaacqWFXeIraaa@3787@(*frag*(*h*, *i*, *j*)) ≤ *minfr*},     (6)

where *h *ranges over all possible fragments. Given a haplotype *H*, the longest matching fragments in ℱ
 MathType@MTEF@5@5@+=feaafiart1ev1aaatCvAUfKttLearuWrP9MDH5MBPbIqV92AaeXatLxBI9gBamrtHrhAL1wy0L2yHvtyaeHbnfgDOvwBHrxAJfwnaebbnrfifHhDYfgasaacH8akY=wiFfYdH8Gipec8Eeeu0xXdbba9frFj0=OqFfea0dXdd9vqai=hGuQ8kuc9pgc9s8qqaq=dirpe0xb9q8qiLsFr0=vr0=vr0dc8meaabaqaciaacaGaaeqabaWaaeGaeaaakeaaimaacqWFXeIraaa@3787@_*minfr *_are then used to estimate the conditional probabilities at each marker *i*:

P(H)=P(H(1))∏i=2,...,ℓP(H(i)|H(si,i−1)),     (7)
 MathType@MTEF@5@5@+=feaafiart1ev1aaatCvAUfKttLearuWrP9MDH5MBPbIqV92AaeXatLxBI9gBaebbnrfifHhDYfgasaacH8akY=wiFfYdH8Gipec8Eeeu0xXdbba9frFj0=OqFfea0dXdd9vqai=hGuQ8kuc9pgc9s8qqaq=dirpe0xb9q8qiLsFr0=vr0=vr0dc8meaabaqaciaacaGaaeqabaqabeGadaaakeaacqWGqbaucqGGOaakcqWGibascqGGPaqkcqGH9aqpcqWGqbaucqGGOaakcqWGibascqGGOaakcqaIXaqmcqGGPaqkcqGGPaqkdaqeqbqaaiabdcfaqjabcIcaOiabdIeaijabcIcaOiabdMgaPjabcMcaPiabcYha8jabdIeaijabcIcaOiabdohaZnaaBaaaleaacqWGPbqAaeqaaOGaeiilaWIaemyAaKMaeyOeI0IaeGymaeJaeiykaKIaeiykaKcaleaacqWGPbqAcqGH9aqpcqaIYaGmcqGGSaalcqGGUaGlcqGGUaGlcqGGUaGlcqGGSaalcqWItecBaeqaniabg+GivdGccqGGSaalcaWLjaGaaCzcamaabmaabaGaeG4naCdacaGLOaGaayzkaaaaaa@5A55@

where *s*_*i *_= min{*s *| *H*(*s*, *i *- 1) ∈ ℱ
 MathType@MTEF@5@5@+=feaafiart1ev1aaatCvAUfKttLearuWrP9MDH5MBPbIqV92AaeXatLxBI9gBamrtHrhAL1wy0L2yHvtyaeHbnfgDOvwBHrxAJfwnaebbnrfifHhDYfgasaacH8akY=wiFfYdH8Gipec8Eeeu0xXdbba9frFj0=OqFfea0dXdd9vqai=hGuQ8kuc9pgc9s8qqaq=dirpe0xb9q8qiLsFr0=vr0=vr0dc8meaabaqaciaacaGaaeqabaWaaeGaeaaakeaaimaacqWFXeIraaa@3787@_*minfr*_}. The order of the Markov chain is thus individually adjusted for each position and each haplotype.

Although both fixed and variable-order Markov models have been extensively studied and used for many applications (see [34] for a review of variable-order Markov models), we are not aware of any previous applications to haplotype reconstruction. There is also a subtle difference between these models and typical applications of Markov chains. The models employed here are *inhomogeneous*, i.e., each marker has its own states and transition probabilities, whereas usually they are not dependent on the location in the sequence. Also unlike typical applications, we are simultaneously modeling two sequences whose entries are observed together as unordered pairs.

Another alternative of building a haplotype probability model from local fragments is to think of a complete haplotype as a mosaic of frequent fragments, originating from different founders or via different coalescence histories. In the segmentation model we consider non-overlapping fragments to be independent, and consequently define the probability of a haplotype to be the product of fragment probabilities:

P(H)=∏(si,ei)∈SP(H(si,ei)),
 MathType@MTEF@5@5@+=feaafiart1ev1aaatCvAUfKttLearuWrP9MDH5MBPbIqV92AaeXatLxBI9gBaebbnrfifHhDYfgasaacH8akY=wiFfYdH8Gipec8Eeeu0xXdbba9frFj0=OqFfea0dXdd9vqai=hGuQ8kuc9pgc9s8qqaq=dirpe0xb9q8qiLsFr0=vr0=vr0dc8meaabaqaciaacaGaaeqabaqabeGadaaakeaacqWGqbaucqGGOaakcqWGibascqGGPaqkcqGH9aqpdaqeqbqaaiabdcfaqjabcIcaOiabdIeaijabcIcaOiabdohaZnaaBaaaleaacqWGPbqAaeqaaOGaeiilaWIaemyzau2aaSbaaSqaaiabdMgaPbqabaGccqGGPaqkcqGGPaqkcqGGSaalaSqaaiabcIcaOiabdohaZnaaBaaameaacqWGPbqAaeqaaSGaeiilaWIaemyzau2aaSbaaWqaaiabdMgaPbqabaWccqGGPaqkcqGHiiIZcqWGtbWuaeqaniabg+Givdaaaa@4C2F@

where *S *= (*s*_1_, *e*_1_), (*s*_2_, *e*_2_),..., (*s*_*n*_, *e*_*n*_) is a segmentation of *H *into consecutive non-overlapping fragments (such that *s*_1 _= 1, *s*_*i *_= *e*_*i *- 1 _+ 1 for all 1 <*i *≤ *n*, and *e*_*n *_= ℓ).

The above formula leaves open the actual segmentation used for each haplotype. As the recombination history of the haplotypes is unknown, we of course have no way of deducing the "correct" segmentation. As a solution, we propose a model which averages over all possible segmentations:

P(H)=∑S∈S∏(si,ei)∈SP(H(si,ei))|S′|,     (8)
MathType@MTEF@5@5@+=feaafiart1ev1aaatCvAUfKttLearuWrP9MDH5MBPbIqV92AaeXatLxBI9gBamrtHrhAL1wy0L2yHvtyaeHbnfgDOvwBHrxAJfwnaebbnrfifHhDYfgasaacH8akY=wiFfYdH8Gipec8Eeeu0xXdbba9frFj0=OqFfea0dXdd9vqai=hGuQ8kuc9pgc9s8qqaq=dirpe0xb9q8qiLsFr0=vr0=vr0dc8meaabaqaciaacaGaaeqabaWaaeGaeaaakeaacqWGqbaucqGGOaakcqWGibascqGGPaqkcqGH9aqpdaWcaaqaamaaqababaWaaebeaeaacqWGqbaucqGGOaakcqWGibascqGGOaakcqWGZbWCdaWgaaWcbaGaemyAaKgabeaakiabcYcaSiabdwgaLnaaBaaaleaacqWGPbqAaeqaaOGaeiykaKIaeiykaKcaleaacqGGOaakcqWGZbWCdaWgaaadbaGaemyAaKgabeaaliabcYcaSiabdwgaLnaaBaaameaacqWGPbqAaeqaaSGaeiykaKIaeyicI4Saem4uamfabeqdcqGHpis1aaWcbaGaem4uamLaeyicI4mcdaGae8NeXpfabeqdcqGHris5aaGcbaGaeiiFaWNaf8NeXpLbauaacqGG8baFaaGaeiilaWIaaCzcaiaaxMaadaqadaqaaiabiIda4aGaayjkaiaawMcaaaaa@64EA@

where S
 MathType@MTEF@5@5@+=feaafiart1ev1aaatCvAUfKttLearuWrP9MDH5MBPbIqV92AaeXatLxBI9gBamrtHrhAL1wy0L2yHvtyaeHbnfgDOvwBHrxAJfwnaebbnrfifHhDYfgasaacH8akY=wiFfYdH8Gipec8Eeeu0xXdbba9frFj0=OqFfea0dXdd9vqai=hGuQ8kuc9pgc9s8qqaq=dirpe0xb9q8qiLsFr0=vr0=vr0dc8meaabaqaciaacaGaaeqabaWaaeGaeaaakeaaimaacqWFse=uaaa@3845@ is the set of all possible segmentations of *H*: S
 MathType@MTEF@5@5@+=feaafiart1ev1aaatCvAUfKttLearuWrP9MDH5MBPbIqV92AaeXatLxBI9gBamrtHrhAL1wy0L2yHvtyaeHbnfgDOvwBHrxAJfwnaebbnrfifHhDYfgasaacH8akY=wiFfYdH8Gipec8Eeeu0xXdbba9frFj0=OqFfea0dXdd9vqai=hGuQ8kuc9pgc9s8qqaq=dirpe0xb9q8qiLsFr0=vr0=vr0dc8meaabaqaciaacaGaaeqabaWaaeGaeaaakeaaimaacqWFse=uaaa@3845@ = {*S *: *H*(*s*, *e*) ∈ ℱ
 MathType@MTEF@5@5@+=feaafiart1ev1aaatCvAUfKttLearuWrP9MDH5MBPbIqV92AaeXatLxBI9gBamrtHrhAL1wy0L2yHvtyaeHbnfgDOvwBHrxAJfwnaebbnrfifHhDYfgasaacH8akY=wiFfYdH8Gipec8Eeeu0xXdbba9frFj0=OqFfea0dXdd9vqai=hGuQ8kuc9pgc9s8qqaq=dirpe0xb9q8qiLsFr0=vr0=vr0dc8meaabaqaciaacaGaaeqabaWaaeGaeaaakeaaimaacqWFXeIraaa@3787@_*minfr *_for all(*s*, *e*) ∈ *S*}, and S′
 MathType@MTEF@5@5@+=feaafiart1ev1aaatCvAUfKttLearuWrP9MDH5MBPbIqV92AaeXatLxBI9gBamrtHrhAL1wy0L2yHvtyaeHbnfgDOvwBHrxAJfwnaebbnrfifHhDYfgasaacH8akY=wiFfYdH8Gipec8Eeeu0xXdbba9frFj0=OqFfea0dXdd9vqai=hGuQ8kuc9pgc9s8qqaq=dirpe0xb9q8qiLsFr0=vr0=vr0dc8meaabaqaciaacaGaaeqabaWaaeGaeaaakeaaimaacuWFse=ugaqbaaaa@3851@ is the set of all possible segmentations of the marker map:

S′
 MathType@MTEF@5@5@+=feaafiart1ev1aaatCvAUfKttLearuWrP9MDH5MBPbIqV92AaeXatLxBI9gBamrtHrhAL1wy0L2yHvtyaeHbnfgDOvwBHrxAJfwnaebbnrfifHhDYfgasaacH8akY=wiFfYdH8Gipec8Eeeu0xXdbba9frFj0=OqFfea0dXdd9vqai=hGuQ8kuc9pgc9s8qqaq=dirpe0xb9q8qiLsFr0=vr0=vr0dc8meaabaqaciaacaGaaeqabaWaaeGaeaaakeaaimaacuWFse=ugaqbaaaa@3851@ = {*S *: for all(*s*, *e*) ∈ *S *there exists some frag(*h*, *s*, *e*) ∈ ℱ
 MathType@MTEF@5@5@+=feaafiart1ev1aaatCvAUfKttLearuWrP9MDH5MBPbIqV92AaeXatLxBI9gBamrtHrhAL1wy0L2yHvtyaeHbnfgDOvwBHrxAJfwnaebbnrfifHhDYfgasaacH8akY=wiFfYdH8Gipec8Eeeu0xXdbba9frFj0=OqFfea0dXdd9vqai=hGuQ8kuc9pgc9s8qqaq=dirpe0xb9q8qiLsFr0=vr0=vr0dc8meaabaqaciaacaGaaeqabaWaaeGaeaaakeaaimaacqWFXeIraaa@3787@_*minfr*_} (note that the normalization factor is independent of the haplotype *H*). The above formula can be interpreted as having a set of all possible block models, each with a uniform prior probability, over which the probability is averaged. Note that in contrast to widely used block models, there are no preferences for shared block boundaries between individuals; instead, all possible segmentations are considered as equally viable alternatives.

A potential weakness of the model defined by Equation 8 is that there are more segmentations with a large number of segments than ones with a smaller number. To favor segmentations with less and longer segments, we introduce a penalty factor (controlled by an additional parameter *q*) for each additional fragment included in the segmentation:

P(H)=∑S∈Sq|S|−1.∏(si,ei)∈SP(H(si,ei))∑S∈S′q|S|−1,     (9)
MathType@MTEF@5@5@+=feaafiart1ev1aaatCvAUfKttLearuWrP9MDH5MBPbIqV92AaeXatLxBI9gBamrtHrhAL1wy0L2yHvtyaeHbnfgDOvwBHrxAJfwnaebbnrfifHhDYfgasaacH8akY=wiFfYdH8Gipec8Eeeu0xXdbba9frFj0=OqFfea0dXdd9vqai=hGuQ8kuc9pgc9s8qqaq=dirpe0xb9q8qiLsFr0=vr0=vr0dc8meaabaqaciaacaGaaeqabaWaaeGaeaaakeaacqWGqbaucqGGOaakcqWGibascqGGPaqkcqGH9aqpdaWcaaqaamaaqababaGaemyCae3aaWbaaSqabeaacqGG8baFcqWGtbWucqGG8baFcqGHsislcqaIXaqmaaGccqGGUaGldaqeqaqaaiabdcfaqjabcIcaOiabdIeaijabcIcaOiabdohaZnaaBaaaleaacqWGPbqAaeqaaOGaeiilaWIaemyzau2aaSbaaSqaaiabdMgaPbqabaGccqGGPaqkcqGGPaqkaSqaaiabcIcaOiabdohaZnaaBaaameaacqWGPbqAaeqaaSGaeiilaWIaemyzau2aaSbaaWqaaiabdMgaPbqabaWccqGGPaqkcqGHiiIZcqWGtbWuaeqaniabg+GivdaaleaacqWGtbWucqGHiiIZimaacqWFse=uaeqaniabggHiLdaakeaadaaeqaqaaiabdghaXnaaCaaaleqabaGaeiiFaWNaem4uamLaeiiFaWNaeyOeI0IaeGymaedaaaqaaiabdofatjabgIGiolqb=jr8tzaafaaabeqdcqGHris5aaaakiabcYcaSiaaxMaacaWLjaWaaeWaaeaacqaI5aqoaiaawIcacaGLPaaaaaa@76B7@

where 0 <*q *≤ 1, and |*S*| is the number of segments in segmentation *S*. Setting a smaller value to *q *will cause segmentations with a large number of fragments to have a smaller probability. Setting *q *= 1 corresponds to no penalty, in which case the formulation is equivalent to Equation 8. By experimenting we found that *q *= 0.1 works reasonably well in different settings. This value was used in all our experiments.

### The HaploRec algorithm

Given one of the models we defined – fixed-order Markov chain, variable-order Markov chain, or the segmentation model – and its parameters, we can compute a probability for any given haplotype.

Assuming independence between the two haplotypes of an individual (*Hardy-Weinberg equilibrium*), the probability of any haplotype configuration for a genotype is just the product of the probabilities of its constituent haplotypes. The algorithmic problem is twofold: the haplotype reconstruction method has to simultaneously learn the model parameters and reconstruct the haplotypes of each individual.

Our algorithm, HaploRec, is a modified version of the the EM algorithm introduced in [4]. In the EM framework, the haplotype configuration underlying each genotype is considered as a latent variable, and the goal is to find a maximum-likelihood estimate for the model parameters ℱ
 MathType@MTEF@5@5@+=feaafiart1ev1aaatCvAUfKttLearuWrP9MDH5MBPbIqV92AaeXatLxBI9gBamrtHrhAL1wy0L2yHvtyaeHbnfgDOvwBHrxAJfwnaebbnrfifHhDYfgasaacH8akY=wiFfYdH8Gipec8Eeeu0xXdbba9frFj0=OqFfea0dXdd9vqai=hGuQ8kuc9pgc9s8qqaq=dirpe0xb9q8qiLsFr0=vr0=vr0dc8meaabaqaciaacaGaaeqabaWaaeGaeaaakeaaimaacqWFXeIraaa@3787@. The likelihood of a single genotype is a sum of the probabilities of all its possible haplotype configurations (the values of latent variables), and the likelihood of the whole data is then just a product over all genotypes:

L(G|ℱ)=∏G∈G∑{H1,H2}∈CGP({H1,H2}|ℱ),     (10)
 MathType@MTEF@5@5@+=feaafiart1ev1aaatCvAUfKttLearuWrP9MDH5MBPbIqV92AaeXatLxBI9gBamrtHrhAL1wy0L2yHvtyaeHbnfgDOvwBHrxAJfwnaebbnrfifHhDYfgasaacH8akY=wiFfYdH8Gipec8Eeeu0xXdbba9frFj0=OqFfea0dXdd9vqai=hGuQ8kuc9pgc9s8qqaq=dirpe0xb9q8qiLsFr0=vr0=vr0dc8meaabaqaciaacaGaaeqabaWaaeGaeaaakeaacqWGmbatcqGGOaakimaacqWFge=rcqGG8baFcqWFXeIrcqGGPaqkcqGH9aqpdaqeqbqaamaaqafabaGaemiuaaLaeiikaGIaei4EaSNaemisaG0aaSbaaSqaaiabigdaXaqabaGccqGGSaalcqWGibasdaWgaaWcbaGaeGOmaidabeaakiabc2ha9jabcYha8jab=ftigjabcMcaPaWcbaGaei4EaSNaemisaG0aaSbaaWqaaiabigdaXaqabaWccqGGSaalcqWGibasdaWgaaadbaGaeGOmaidabeaaliabc2ha9jabgIGiolabdoeadnaaBaaameaacqWGhbWraeqaaaWcbeqdcqGHris5aaWcbaGaem4raCKaeyicI4Sae8NbXFeabeqdcqGHpis1aOGaeiilaWIaaCzcaiaaxMaadaqadaqaaiabigdaXiabicdaWaGaayjkaiaawMcaaaaa@66E9@

where *P*({*H*_1_, *H*_2_} | ℱ
 MathType@MTEF@5@5@+=feaafiart1ev1aaatCvAUfKttLearuWrP9MDH5MBPbIqV92AaeXatLxBI9gBamrtHrhAL1wy0L2yHvtyaeHbnfgDOvwBHrxAJfwnaebbnrfifHhDYfgasaacH8akY=wiFfYdH8Gipec8Eeeu0xXdbba9frFj0=OqFfea0dXdd9vqai=hGuQ8kuc9pgc9s8qqaq=dirpe0xb9q8qiLsFr0=vr0=vr0dc8meaabaqaciaacaGaaeqabaWaaeGaeaaakeaaimaacqWFXeIraaa@3787@) = *P*(*H*_1_|ℱ
 MathType@MTEF@5@5@+=feaafiart1ev1aaatCvAUfKttLearuWrP9MDH5MBPbIqV92AaeXatLxBI9gBamrtHrhAL1wy0L2yHvtyaeHbnfgDOvwBHrxAJfwnaebbnrfifHhDYfgasaacH8akY=wiFfYdH8Gipec8Eeeu0xXdbba9frFj0=OqFfea0dXdd9vqai=hGuQ8kuc9pgc9s8qqaq=dirpe0xb9q8qiLsFr0=vr0=vr0dc8meaabaqaciaacaGaaeqabaWaaeGaeaaakeaaimaacqWFXeIraaa@3787@)·*P*(*H*_2_|ℱ
 MathType@MTEF@5@5@+=feaafiart1ev1aaatCvAUfKttLearuWrP9MDH5MBPbIqV92AaeXatLxBI9gBamrtHrhAL1wy0L2yHvtyaeHbnfgDOvwBHrxAJfwnaebbnrfifHhDYfgasaacH8akY=wiFfYdH8Gipec8Eeeu0xXdbba9frFj0=OqFfea0dXdd9vqai=hGuQ8kuc9pgc9s8qqaq=dirpe0xb9q8qiLsFr0=vr0=vr0dc8meaabaqaciaacaGaaeqabaWaaeGaeaaakeaaimaacqWFXeIraaa@3787@) is the probability of the haplotype pair {*H*_1_, *H*_2_}, given the model parameters ℱ
 MathType@MTEF@5@5@+=feaafiart1ev1aaatCvAUfKttLearuWrP9MDH5MBPbIqV92AaeXatLxBI9gBamrtHrhAL1wy0L2yHvtyaeHbnfgDOvwBHrxAJfwnaebbnrfifHhDYfgasaacH8akY=wiFfYdH8Gipec8Eeeu0xXdbba9frFj0=OqFfea0dXdd9vqai=hGuQ8kuc9pgc9s8qqaq=dirpe0xb9q8qiLsFr0=vr0=vr0dc8meaabaqaciaacaGaaeqabaWaaeGaeaaakeaaimaacqWFXeIraaa@3787@. The EM algorithm works by iteratively improving estimates of the model parameters (*the M-step*) and values of the latent variables (*the E-step*), until a (local) maximum of the likelihood is reached. The individual reconstructed haplotypes can then be obtained by just selecting, for each genotype, the compatible haplotype pair that has maximal probability according to the obtained maximum-likelihood estimate of the parameters.

The original algorithm [4] uses a multinomial model, where parameters are the frequencies of complete haplotypes. Our modifications to the EM algorithm consist of (1) replacing the multinomial model with one of our fragment-based probability models and (2) using a sequential pruning strategy to overcome the exponential computational complexity of the E-step. Below, we give an outline of the HaploRec algorithm; a more detailed description, including pseudo-code, handling of missing data and complexity analysis, is given in Additional file 1.

#### Representation of model parameters

Parameters of the three models are slightly different. In the Markov models, the parameters are the conditional allele probabilities; in the segmentation model, the parameters are the fragment probabilities. In practice, the conditional probabilities are derived from fragment probabilities as follows:

P(H(i)|H(i−d,i−1))=ℱ(H(i−d,i))ℱ(H(i−d,i−1)),
 MathType@MTEF@5@5@+=feaafiart1ev1aaatCvAUfKttLearuWrP9MDH5MBPbIqV92AaeXatLxBI9gBamrtHrhAL1wy0L2yHvtyaeHbnfgDOvwBHrxAJfwnaebbnrfifHhDYfgasaacH8akY=wiFfYdH8Gipec8Eeeu0xXdbba9frFj0=OqFfea0dXdd9vqai=hGuQ8kuc9pgc9s8qqaq=dirpe0xb9q8qiLsFr0=vr0=vr0dc8meaabaqaciaacaGaaeqabaWaaeGaeaaakeaacqWGqbaucqGGOaakcqWGibascqGGOaakcqWGPbqAcqGGPaqkcqGG8baFcqWGibascqGGOaakcqWGPbqAcqGHsislcqWGKbazcqGGSaalcqWGPbqAcqGHsislcqaIXaqmcqGGPaqkcqGGPaqkcqGH9aqpdaWcaaqaaGWaaiab=ftigjabcIcaOiabdIeaijabcIcaOiabdMgaPjabgkHiTiabdsgaKjabcYcaSiabdMgaPjabcMcaPiabcMcaPaqaaiab=ftigjabcIcaOiabdIeaijabcIcaOiabdMgaPjabgkHiTiabdsgaKjabcYcaSiabdMgaPjabgkHiTiabigdaXiabcMcaPiabcMcaPaaacqGGSaalaaa@6416@

where *d *is the number of previous markers conditioned on and ℱ
 MathType@MTEF@5@5@+=feaafiart1ev1aaatCvAUfKttLearuWrP9MDH5MBPbIqV92AaeXatLxBI9gBamrtHrhAL1wy0L2yHvtyaeHbnfgDOvwBHrxAJfwnaebbnrfifHhDYfgasaacH8akY=wiFfYdH8Gipec8Eeeu0xXdbba9frFj0=OqFfea0dXdd9vqai=hGuQ8kuc9pgc9s8qqaq=dirpe0xb9q8qiLsFr0=vr0=vr0dc8meaabaqaciaacaGaaeqabaWaaeGaeaaakeaaimaacqWFXeIraaa@3787@(*H*) denotes the estimated probability (frequency) of fragment *H*. As conditional probabilities can be straightforwardly derived from fragment frequencies, we always use the set of fragments as a representation for the model parameters, also in the case of the Markov models.

#### E-step

In the E-step of the EM algorithm, the aim is to calculate, for each *G *∈ G
 MathType@MTEF@5@5@+=feaafiart1ev1aaatCvAUfKttLearuWrP9MDH5MBPbIqV92AaeXatLxBI9gBamrtHrhAL1wy0L2yHvtyaeHbnfgDOvwBHrxAJfwnaebbnrfifHhDYfgasaacH8akY=wiFfYdH8Gipec8Eeeu0xXdbba9frFj0=OqFfea0dXdd9vqai=hGuQ8kuc9pgc9s8qqaq=dirpe0xb9q8qiLsFr0=vr0=vr0dc8meaabaqaciaacaGaaeqabaWaaeGaeaaakeaaimaacqWFge=raaa@382D@, and each compatible haplotype pair {*H*_1_, *H*_2_} ∈ *C*_*G*_, the probability that the genotype actually consists of that haplotype pair:

Pt({H1,H2}|G)=P({H1,H2}|G,ℱt−1)=P(H1|ℱt−1)P(H2|ℱt−1)∑{H,H¯}∈CGP(H|ℱt−1)P(H¯|ℱt−1).
 MathType@MTEF@5@5@+=feaafiart1ev1aaatCvAUfKttLearuWrP9MDH5MBPbIqV92AaeXatLxBI9gBamrtHrhAL1wy0L2yHvtyaeHbnfgDOvwBHrxAJfwnaebbnrfifHhDYfgasaacH8akY=wiFfYdH8Gipec8Eeeu0xXdbba9frFj0=OqFfea0dXdd9vqai=hGuQ8kuc9pgc9s8qqaq=dirpe0xb9q8qiLsFr0=vr0=vr0dc8meaabaqaciaacaGaaeqabaWaaeGaeaaakeaacqWGqbaudaWgaaWcbaGaemiDaqhabeaakiabcIcaOiabcUha7jabdIeainaaBaaaleaacqaIXaqmaeqaaOGaeiilaWIaemisaG0aaSbaaSqaaiabikdaYaqabaGccqGG9bqFcqGG8baFcqWGhbWrcqGGPaqkcqGH9aqpcqWGqbaucqGGOaakcqGG7bWEcqWGibasdaWgaaWcbaGaeGymaedabeaakiabcYcaSiabdIeainaaBaaaleaacqaIYaGmaeqaaOGaeiyFa0NaeiiFaWNaem4raCKaeiilaWccdaGae8xmHy0aaSbaaSqaaiabdsha0jabgkHiTiabigdaXaqabaGccqGGPaqkcqGH9aqpdaWcaaqaaiabdcfaqjabcIcaOiabdIeainaaBaaaleaacqaIXaqmaeqaaOGaeiiFaWNae8xmHy0aaSbaaSqaaiabdsha0jabgkHiTiabigdaXaqabaGccqGGPaqkcqWGqbaucqGGOaakcqWGibasdaWgaaWcbaGaeGOmaidabeaakiabcYha8jab=ftignaaBaaaleaacqWG0baDcqGHsislcqaIXaqmaeqaaOGaeiykaKcabaWaaabeaeaacqWGqbaucqGGOaakcqWGibascqGG8baFcqWFXeIrdaWgaaWcbaGaemiDaqNaeyOeI0IaeGymaedabeaakiabcMcaPiabdcfaqjabcIcaOiqbdIeaizaaraGaeiiFaWNae8xmHy0aaSbaaSqaaiabdsha0jabgkHiTiabigdaXaqabaGccqGGPaqkaSqaaiabcUha7jabdIeaijabcYcaSiqbdIeaizaaraGaeiyFa0NaeyicI4Saem4qam0aaSbaaWqaaiabdEeahbqabaaaleqaniabggHiLdaaaOGaeiOla4caaa@92E9@

The model parameters, ℱ
 MathType@MTEF@5@5@+=feaafiart1ev1aaatCvAUfKttLearuWrP9MDH5MBPbIqV92AaeXatLxBI9gBamrtHrhAL1wy0L2yHvtyaeHbnfgDOvwBHrxAJfwnaebbnrfifHhDYfgasaacH8akY=wiFfYdH8Gipec8Eeeu0xXdbba9frFj0=OqFfea0dXdd9vqai=hGuQ8kuc9pgc9s8qqaq=dirpe0xb9q8qiLsFr0=vr0=vr0dc8meaabaqaciaacaGaaeqabaWaaeGaeaaakeaaimaacqWFXeIraaa@3787@_*t *- 1_, are obtained from the previous parameter estimation step. The normalization just transforms the (prior) probabilities given by the model into a (posterior) probability distribution for configurations of the single genotype *G*. Exhaustively going through all possible configurations is feasible only when the number of heterozygous markers is small (≅ 20 or less). With more markers, we use a pruning strategy in which the set of possible haplotype configurations (separately for each genotype) is built up marker by marker, starting from a partial configuration containing only the allele pair at the leftmost marker. At each step, all partial configurations are extended with the allele pair at the next marker (for homozygous markers, each configuration is extended with the same allele pair; for heterozygous markers, there are two possible extensions for each configuration, and only the the *B *most probable configurations are propagated to the next step). In the final step, the *C*(≤ *B*) most probable configurations are returned. The approach is greedy; it is not guaranteed that the set of returned configurations consists exactly of the most probable ones. In preliminary experiments it was found that *B *= 25 and *C *= 10 give a reasonable computational efficiency, while increasing the parameters beyond these values does not significantly improve accuracy. These values were used in the experiments. Using a sequential pruning strategy to implement the E-step is conceptually simple, and has already been used in [19]. However, implementing it efficiently for our models is not trivial. In Additional file 1, we provide a detailed description of the data structures and algorithms for implementing the E-step for each of proposed models.

#### M-step

In the M-step of the EM algorithm, the model parameters are re-estimated (based on the current haplotype estimates from the previous E-step), such that the likelihood of the data is improved from the previous iteration. The haplotype estimates from the previous E-step give the expected frequency of any haplotype in a given genotype. The estimated frequency of a haplotype fragment in a single genotype is obtained by a sum over all the haplotypes in its set of possible configurations that match the fragment, and the overall estimated frequency of a fragment *h *is obtained as an average over all genotypes:

ℱt(h)=12|G|∑G∈G∑{H1,H2}∈CGPt({H1,H2}|G)δh,{H1,H2},
 MathType@MTEF@5@5@+=feaafiart1ev1aaatCvAUfKttLearuWrP9MDH5MBPbIqV92AaeXatLxBI9gBamrtHrhAL1wy0L2yHvtyaeHbnfgDOvwBHrxAJfwnaebbnrfifHhDYfgasaacH8akY=wiFfYdH8Gipec8Eeeu0xXdbba9frFj0=OqFfea0dXdd9vqai=hGuQ8kuc9pgc9s8qqaq=dirpe0xb9q8qiLsFr0=vr0=vr0dc8meaabaqaciaacaGaaeqabaWaaeGaeaaakeaaimaacqWFXeIrdaWgaaWcbaGaemiDaqhabeaakiabcIcaOiabdIgaOjabcMcaPiabg2da9maalaaabaGaeGymaedabaGaeGOmaiJaeiiFaWNae8NbXFKaeiiFaWhaamaaqafabaWaaabuaeaacqWGqbaudaWgaaWcbaGaemiDaqhabeaaaeaacqGG7bWEcqWGibasdaWgaaadbaGaeGymaedabeaaliabcYcaSiabdIeainaaBaaameaacqaIYaGmaeqaaSGaeiyFa0NaeyicI4Saem4qam0aaSbaaWqaaiabdEeahbqabaaaleqaniabggHiLdaaleaacqWGhbWrcqGHiiIZcqWFge=raeqaniabggHiLdGccqGGOaakcqGG7bWEcqWGibasdaWgaaWcbaGaeGymaedabeaakiabcYcaSiabdIeainaaBaaaleaacqaIYaGmaeqaaOGaeiyFa0NaeiiFaWNaem4raCKaeiykaKccciGae4hTdq2aaSbaaSqaaiabdIgaOjabcYcaSiabcUha7jabdIeainaaBaaameaacqaIXaqmaeqaaSGaeiilaWIaemisaG0aaSbaaWqaaiabikdaYaqabaWccqGG9bqFaeqaaOGaeiilaWcaaa@75AA@

where δh,{H1,H2}
 MathType@MTEF@5@5@+=feaafiart1ev1aaatCvAUfKttLearuWrP9MDH5MBPbIqV92AaeXatLxBI9gBamrtHrhAL1wy0L2yHvtyaeHbnfgDOvwBHrxAJfwnaebbnrfifHhDYfgasaacH8akY=wiFfYdH8Gipec8Eeeu0xXdbba9frFj0=OqFfea0dXdd9vqai=hGuQ8kuc9pgc9s8qqaq=dirpe0xb9q8qiLsFr0=vr0=vr0dc8meaabaqaciaacaGaaeqabaWaaeGaeaaakeaaiiGacqWF0oazdaWgaaWcbaGaemiAaGMaeiilaWIaei4EaSNaemisaG0aaSbaaWqaaiabigdaXaqabaWccqGGSaalcqWGibasdaWgaaadbaGaeGOmaidabeaaliabc2ha9bqabaaaaa@42D4@ = |{*i *∈ {1, 2} | *h *matches *H*_*i*_}| ∈ {0, 1, 2} is the number of haplotypes in configuration {*H*_1_, *H*_2_} that match *h*. To compute the fragment frequencies, we first combine the sets of most probable haplotype configurations (from the previous E-step) from all genotypes into a weighted (multi-)set of haplotypes, where the weight of each haplotype is the sum of the probabilities of its occurrences in the reconstructed configurations (note that the weights are thus mostly non-integer). Computing the set of frequent fragments is then done by by depth-first search in the fragment containment lattice. First, all possible fragments of length one are generated, after which they are recursively extended to the right as long as the frequencies of resulting fragments stay above the given minimum frequency threshold and the fragments do not exceed a given maximum length. To improve the efficiency of the algorithm, the list of matching haplotypes is stored with each fragment during the execution of the algorithm (as depth-first search is used, this does not significantly improve memory usage, because the list can be freed when the fragment is no longer in the stack). This way, the frequency of a each fragment can be computed by only matching its last allele to the list of haplotypes matching its prefix. The algorithm is guaranteed to find all frequent fragments, as frequency decreases monotonically when a fragment is extended.

#### Initialization

The initial frequencies are computed somewhat similarly as in the parameter estimation step. The difference is that there is not yet any information about the probability of different haplotype configurations, and thus all configurations of a genotype must be considered equally likely. The number of haplotype configurations compatible with a genotype *G *is exponential in the number of heterozygous markers in *G*, and enumerating all the possible configurations in *C*_*G *_is thus infeasible. Fortunately, the initial frequencies can be counted directly from the genotype data as follows, without explicitly generating the elements of *C*_*G*_:

ℱ(frag(h,i,j))=12|G|∑G∈G,G matches frag(h,i,j)21−kG(i,j),     (11)
 MathType@MTEF@5@5@+=feaafiart1ev1aaatCvAUfKttLearuWrP9MDH5MBPbIqV92AaeXatLxBI9gBamrtHrhAL1wy0L2yHvtyaeHbnfgDOvwBHrxAJfwnaebbnrfifHhDYfgasaacH8akY=wiFfYdH8Gipec8Eeeu0xXdbba9frFj0=OqFfea0dXdd9vqai=hGuQ8kuc9pgc9s8qqaq=dirpe0xb9q8qiLsFr0=vr0=vr0dc8meaabaqaciaacaGaaeqabaWaaeGaeaaakeaaimaacqWFXeIrcqGGOaakcqWGMbGzcqWGYbGCcqWGHbqycqWGNbWzcqGGOaakcqWGObaAcqGGSaalcqWGPbqAcqGGSaalcqWGQbGAcqGGPaqkcqGGPaqkcqGH9aqpdaWcaaqaaiabigdaXaqaaiabikdaYiabcYha8jab=zq8hjabcYha8baadaaeqbqaaiabikdaYmaaCaaaleqabaGaeGymaeJaeyOeI0Iaem4AaS2aaSbaaWqaaiabdEeahjabcIcaOiabdMgaPjabcYcaSiabdQgaQjabcMcaPaqabaaaaaWcbaGaem4raCKaeyicI4Sae8NbXFKaeiilaWIaem4raCKaeeiiaaIaeeyBa0MaeeyyaeMaeeiDaqNaee4yamMaeeiAaGMaeeyzauMaee4CamNaeeiiaaIaemOzayMaemOCaiNaemyyaeMaem4zaCMaeiikaGIaemiAaGMaeiilaWIaemyAaKMaeiilaWIaemOAaOMaeiykaKcabeqdcqGHris5aOGaeiilaWIaaCzcaiaaxMaadaqadaqaaiabigdaXiabigdaXaGaayjkaiaawMcaaaaa@7EC3@

where *k*_*G*(*i*, *j*) _is the number of heterozygous markers in genotype fragment *G*(*i*, *j*) (note that a homozygous genotype has two identical haplotypes both matching the fragment, and thus weight 2). Computing the initial model can be implemented by a slightly modified version of the above-described depth-first search algorithm.

### Using overlapping windows to haplotype very long marker maps

The HaploRec algorithm described above is directly suitable for windows (marker maps) containing up to 500–1500 markers. This practical limit depends on the number of genotypes, amount of missing data, complexity of the haplotype distribution, program parameters and the amount of available main memory. We have extended the implementation of the method by a simple but efficient feature that allows it to handle an arbitrarily large number of markers. Briefly, the idea is to sequentially haplotype a window of *w *markers at a time. In each window except the first one, phased haplotypes for the first *o *markers are obtained from the haplotypes in the previous, overlapping window. After a window is haplotyped, *r *of its last markers are discarded; they are only used to get better estimates for the *w *- *o *- *r *markers in the middle of the window. I.e., each window has length *w *= *o *+ *u *+ *o *+ *r*, where *u *is the number of markers unique to this window, the first *o *represents the haplotypes obtained from the previous window and the second *o *those that are used by the next window. The window is moved *o *+ *u *markers at a time. In experiments with Yoruba data (results not shown), HAPLOREC had practically identical accuracies with this extension and without it, at the expense of slightly larger running times per marker resulting from the overlapping windows.

### Simulation of data

We used Hudson's coalescence simulator [24] to generate chromosomes under the standard Wright-Fisher neutral model of genetic variation with recombination. A long chromosomal region with 16666666 base pairs was simulated. The probability of a mutation in each base pair was set to 10^-8 ^per generation, and the probability of cross-over between adjacent base pairs was set to 10^-8^. These values give the mutation probability for the entire chromosomal region *μ *= 0.16666666, and cross-over probability *ρ *= 0.16666665. The diploid population size, *N*_0_, was set to the standard 10000, giving the mutation parameter *θ *= 4*N*_0_*μ *= 6666.6666, and the recombination parameter *r *= 6666.6665. This standard model simulates recombinations with a uniform distribution. As a result of the stochastic coalescence, however, the recombinations in the final population tend not to be uniformly distributed.

A sample of 2000 chromosomes was generated, and these were paired to form 1000 genotypes. On the average, one simulation produced approximately 55000 segregating sites. Markers were chosen from the segregating sites with minor allele frequency at least 5%, such that marker spacing was as uniform as possible. The actual number of markers and the position of the markers varied with different test settings. The amount of linkage disequilibrium between markers is perhaps the most important factor affecting the accuracy of population-based haplotyping methods. Under the assumption of uniform recombination the amount of linkage disequilibrium is governed by the average distance between adjacent markers. To assess haplotyping performance with different amounts of linkage disequilibrium, marker spacing was varied between 6.6 and 166 kb. The average linkage disequilibrium between neighboring markers, measured with Lewontins |*D'*| measure, ranges respectively from 0.88 to 0.36 (Table 1). With 30 markers (the number of markers most commonly used in the following experiments), total map lengths are between 200 and 5000 kb. The largest data sets have 500 markers picked from the whole simulated region, giving an average marker spacing of 33 kb.

In real data, a fraction of alleles is practically always missing, and there may be genotyping errors. Therefore, in some of the experiments, part of the alleles were masked as missing. Either both or none of the homologous alleles of each marker were masked. This was done by fixing for each allele pair (single marker of a single genotype) a probability for having missing data. Likewise, genotyping errors were simulated by randomly changing each allele according to a given probability.

### Processing of HapMap data

We used 30 trios from the Yoruba population in Ibadan, Nigeria, and another 30 trios from the CEPH population. Both data sets were downloaded from the HapMap web site [25].

The haplotypes of the children were inferred from the trios, and the non-transmitted parental chromosomes of each trio were combined to form additional artificial haplotype pairs (as is common in association studies if trios are available), resulting in a set of 60 genotypes for each population. Markers for which the phase could not be inferred, when all members of the trio were heterozygous, are included in the resulting data sets, but are not used in the switch accuracy calculations. In markers where only one allele was missing, the other was marked missing as well, since some of the tested programs could not handle markers where only one allele is missing. Markers with minor allele frequency less than 5% were discarded.

For testing, we first sampled 50 sets of markers from distinct regions of chromosome 1. The sampling was done by systematically taking (from the set of markers fulfilling the minor allele frequency threshold) markers 1–500 to the first data set, markers 501–1000 to the second, etc. In the resulting data sets, the average marker spacing is approximately 1.5 kb and the fraction of missing alleles is approximately 3.6%. Data sets with sparser maps were obtained by sampling markers from these; e.g., an average distance of 6 kb between markers was obtained by systematically choosing every fourth marker from the original samples.

For experiments with different numbers of markers, the markers were picked from the middle of each set of 500 markers. To reduce variance in results, caused by differences in the difficulty of haplotyping different markers, accuracy was always evaluated only on the 10 middlemost markers common to all the sets. This way, in each run, the test is the same and results are better comparable.

The same procedures were performed separately for the Yoruba and CEPH data sets, and the same set of experiments were performed for both populations. For each population, the reported experimental results are averaged over the 50 data sets to reduce variance in the results.

## Availability and requirements

• **Project name: **HaploRec

• **Project home page: **.

• **Operating system: **Platform independent

• **Programming language: **Java

• **Other requirements: **Java 1.5 or higher

• **License: **Free for educational, research and non-profit purposes

• **Any restrictions to use by non-academics: **License required

## Authors' contributions

All authors contributed to the formulation of the models and to drafting of the manuscript. LE developed the algorithms and carried out the experiments. FG participated in the design of the study. HT conceived the study and participated in its design. All authors read and approved the final manuscript.

## Supplementary Material

Additional file 1 Detailed algorithms, proof of convergence, and complexity analysis. Click here for file

Additional file 2 Example results from the 10 data replicates. Click here for file
