# Supplementary material for: HaploRec: efficient and accurate large-scale reconstruction of haplotypes
Source: BMC Bioinformatics. 2006 Dec 22;7:542. doi: 10.1186/1471-2105-7-542 (PMC1766938; doi:10.1186/1471-2105-7-542)
Supplement: Additional file 1 — Detailed algorithms, proof of convergence, and complexity analysis. [file 1471-2105-7-542-S1.pdf]

Additional file 1: Algorithms, proof of convergence, and complexity analysis

1 Algorithms

Given one of the models we defined — fixed-order Markov chain, variable-order Markov chain, or the segmentation model — and its parameters, we can in principle estimate the probability of any given haplotype. Consequently, assuming independence between the two haplotypes of an individual (*Hardy-Weinberg equilibrium*), we can estimate the probability of any haplotype configuration of any genotype. We reproduce the central equations here for reference.

Fixed-order Markov model:

$$P(H) = P(H(1,d)) \prod_{i=d+1,\ldots,\ell} P(H(i) \mid H(i-d, i-1)) \tag{1}$$

Collection of frequent fragments:

$$\mathcal{F}_{minfr} = \{frag(h,i,j) \mid 1 \leq i \leq j \leq \ell, \mathcal{F}(frag(h,i,j)) \geq minfr\}, \tag{2}$$

Variable-order Markov model:

$$P(H) = P(H(1)) \prod_{i=2,\ldots,\ell} P(H(i) \mid H(s_i, i-1)) \tag{3}$$

Segmentation model:

$$P(H) = \frac{\sum_{S \in \mathcal{S}} q^{|S|-1} \cdot \prod_{(s_i,e_i) \in S} P(H(s_i,e_i))}{\sum_{S \in \mathcal{S}'} q^{|S|-1}} \tag{4}$$

The algorithmic problem is twofold: the haplotype reconstruction method has to simultaneously learn the model parameters and reconstruct the individual haplotypes. Our haplotype reconstruction algorithm,HaploRec, is based on maximum likelihood estimation with the EM algorithm [1, 2]. For finding frequent fragments and computing their frequencies in the M-step of the algorithm, we use depth-first search. In the E-step of the algorithm, we use a sequential pruning approach to avoid the computational complexity of enumerating all possible haplotype configurations. Efficient computation of the models during the E-step is also crucial for the practical applicability of the method. In the following, we describe each of these components of the HaploRec algorithm in detail.

Algorithm 1 HAPLOREC($\mathcal{G}, P, \epsilon, B, C, minfr, maxlen$)

1: $\{\mathcal{H}_t$ is the set of reconstructed haplotypes in step $t\}$
2: $\{weight_t(H)$ is the frequency of haplotype H in $\mathcal{H}_t\}$
3: {Parameter initialization step:}
4: $\mathcal{F}_0$:=COMPUTEFREQUENTFRAGMENTS($\mathcal{G}$, [Equation 6], *minfr*, *maxlen*);
5: $L_0 := -\infty$; $\Delta L := \infty$; $t := 1$;
6: **while** $\Delta L > \epsilon$ **do**
7: $\quad \mathcal{H}_t := \emptyset$;
8: $\quad weight_t(H) := 0$ for all H;
9: $\quad$ {Haplotype reconstruction step:}
10: $\quad$ **for all** $G \in \mathcal{G}$ **do**
11: $\quad\quad C_G^t$ = RECONSTRUCTONEGENOTYPE($G, P, \mathcal{F}_{t-1}, B, C$);
12: $\quad\quad$ **for all** $\{H_1, H_2\} \in C_G^t$ **do**
13: $\quad\quad\quad P_t(\{H_1, H_2\} \mid G) := \frac{P_t(\{H_1,H_2\})}{\sum_{\{H,\overline{H}\}\in C_G^t} P_t(\{H,\overline{H}\})}$;
14: $\quad\quad\quad \mathcal{H}_t := \mathcal{H}_t \cup \{H_1\} \cup \{H_2\}$;
15: $\quad\quad\quad weight_t(H_1) := weight(H_1) + \frac{1}{2|\mathcal{G}|} P_t(\{H_1, H_2\} \mid G)$;
16: $\quad\quad\quad weight_t(H_2) := weight(H_2) + \frac{1}{2|\mathcal{G}|} P_t(\{H_1, H_2\} \mid G)$;
17: $\quad\quad$ **end for**
18: $\quad$ **end for**
19: $\quad$ {Parameter estimation step:}
20: $\quad \mathcal{F}_t$ =COMPUTEFREQUENTFRAGMENTS($\mathcal{H}_t$, $weight_t$, *minfr*, *maxlen*);
21: $\quad$ Compute the likelihood L_t according to Eq. 5;
22: $\quad \Delta L := L_t - L_{t-1}$; $t := t + 1$;
23: **end while**

The overall structure of HaploRec

HAPLOREC (Algorithm 1), is a modified version of the EM algorithm. The algorithm aims to find a maximum-likelihood estimate for the model parameters $\mathcal{F}$, by iteratively improving estimates for (1) the model parameters (lines 4 and 20) and (2) the haplotypes of each individual (line 13), where the likelihood is defined as

$$L(\mathcal{G} \mid \mathcal{F}) = \prod_{G\in\mathcal{G}} \sum_{\{H_1,H_2\}\in C_G} P(\{H_1, H_2\} \mid \mathcal{F}). \tag{5}$$

HAPLOREC terminates when the change between consecutive likelihoods, ΔL, is smaller than the parameter ϵ (line 6). Our modifications to the EM algorithm [2] consist of (a) a sequential pruning strategy, used to reduce the number of haplotype configurations considered, and (b) the use of fragment-based haplotype probability models instead of a multinomial model.

The sequential pruning strategy is implemented by the subroutine RECONSTRUCTONEGENOTYPE (line 11). This routine enumerates the haplotype configurations of each genotype and computes their probabilities, given a haplotype probability model (P) and the current estimate of the haplotype fragment probabilities ($\mathcal{F}_t$). When the number of markers is large, it is not practical to go through all possible

haplotype configurations, and ReconstructOneGenotype will only return a restricted number of the most probable configurations, with parameters B and C controlling the pruning. We explain the pruning strategy in more detail below.

Parameters of the three models are slightly different. In the Markov models, the parameters are the conditional allele probabilities; in the segmentation model, the parameters are the fragment probabilities. In practice, the conditional probabilities are derived from fragment probabilities as follows:

$$P(H(i) \mid H(i-d, i-1)) = \frac{\mathcal{F}(H(i-d, i))}{\mathcal{F}(H(i-d, i-1))},$$

where d is the number of previous markers conditioned on and $\mathcal{F}(H)$ denotes the estimated probability (frequency) of fragment H. As conditional probabilities can be straightforwardly derived from fragment frequencies, we always use the set of fragments as a representation for the model parameters, also in the case of the Markov models. The fragment frequencies are re-estimated at each parameter estimation step (line 20). Subroutine ComputeFrequentFragments is used to implement this and is controlled by parameters *minfr* and *maxlen*. We explain this in more detail later, too.

We start by explaining the EM-steps performed by HaploRec.

Expectation-Maximization

HaploRec performs at each iteration t (loop between lines 6 and 23), the following two steps:

Haplotype reconstruction step In this step (line 13 in HaploRec) we calculate, for each $G \in \mathcal{G}$, and each compatible haplotype pair $\{H_1, H_2\} \in C_G$, the probability that the genotype actually consists of that haplotype pair:

$$P_t(\{H_1, H_2\} \mid G) = P(\{H_1, H_2\} \mid G, \mathcal{F}_{t-1}) = \frac{P(H_1 \mid \mathcal{F}_{t-1})P(H_2 \mid \mathcal{F}_{t-1})}{\sum_{\{H, \overline{H}\} \in C_G} P(H \mid \mathcal{F}_{t-1})P(\overline{H} \mid \mathcal{F}_{t-1})}.$$

The model parameters, $\mathcal{F}_{t-1}$, are obtained from the previous parameter estimation step. The denominator is just a normalization factor.

Parameter estimation step In this step (line 20 in HaploRec), fragment frequencies $\mathcal{F}_t$ are estimated, conditioned on the previous resolution probabilities. The resolution probabilities from the haplotype reconstruction step give the expected frequency of any haplotype in a given genotype. In a given genotype, the frequency of a haplotype fragment is thus obtained by a sum over all those haplotypes that match the fragment. Finally, the overall frequency of a fragment is obtained as an average over all genotypes:

$$\mathcal{F}_t(h) = \frac{1}{2|\mathcal{G}|} \sum_{G \in \mathcal{G}} \sum_{\{H_1, H_2\} \in C_G} P_t(\{H_1, H_2\} \mid G)\delta_{h, \{H_1, H_2\}},$$

where $\delta_{h,\{H_1,H_2\}} = |\{i \in \{1,2\} \mid h \text{ matches } H_i\}| \in \{0,1,2\}$ is the number of haplotypes in configuration $\{H_1, H_2\}$ that match h.

Parameter initialization step The initial frequencies, $\mathcal{F}_0$, are computed somewhat similarly as in the parameter estimation step. The difference is that there is not yet any information about the probability of different haplotype configurations, and thus all configurations of a genotype must be considered equally likely. The number of haplotype configurations compatible with a genotype G is exponential in the number of heterozygous markers in G, and enumerating all the possible configurations in C_G is thus infeasible. Fortunately, the initial frequencies can be counted directly from the genotype data as follows, without explicitly generating the elements of C_G:

$$\mathcal{F}(\mathit{frag}(h,i,j)) = \frac{1}{2|\mathcal{G}|} \sum_{G \in \mathcal{G}, G \text{ matches } \mathit{frag}(h,i,j)} 2^{1-k_{G(i,j)}}, \tag{6}$$

where $k_{G(i,j)}$ is the number of heterozygous markers in genotype fragment $G(i,j)$ (note that a homozygous genotype has two identical haplotypes both matching the fragment, and thus weight 2).

Convergence As already observed above, HaploRec terminates as soon as no substantial changes occur between consecutive computed likelihoods (this is represented by the condition $\Delta L > \epsilon$ on line 7 in HaploRec). The EM algorithm is guaranteed to converge to a local maximum of the likelihood, as long as the M-step always improves the likelihood from the previous iteration. When using the fixed-order Markov model (Equation 1), the frequency-counting procedure used to estimate the model parameters always finds the maximum-likelihood values for the model parameters (conditioned on the haplotype estimates from the previous E-step), assuming that all haplotype configurations are enumerated in the haplotype reconstruction step, i.e., without pruning (see section Proof of convergence below).

In general, however, we cannot guarantee the convergence to a local maximum. For the variable-order Markov model the context lengths can be different at each iteration, and for the segmentation model, the set of possible segmentations varies between iterations. For these more complex models, it is not theoretically guaranteed that the frequency-counting procedure that is used to update the parameters in the M-step improves the likelihood from the previous iteration. In addition, with a large number of markers, pruning the set of enumerated haplotype configurations is necessary, which further complicates the analysis. In these cases the algorithm terminates when the likelihood would decrease for the first time. However, our experiments with a wide range of different data sets indicate that the algorithm performs well in practice despite this theoretical drawback.

Subroutine ComputeFrequentFragments

The parameter estimation step and the parameter initialization step are both implemented by depth-first search in the fragment containment lattice. Given a set of input haplotypes $\mathcal{H}$ and a weighting function *weight(H)* (the estimated frequency of H), subroutine ComputeFrequentFragments finds all the fragments with frequency at least *minfr* and length at most *maxlen*. Note that the same subroutine can be used to find the set of frequent fragments (Equation 2) used by the variable-order Markov model (Equation 3) and the segmentation model (Equation 4), as well as the set of patterns with fixed lengths d and $d+1$ used by the fixed-order Markov model (Equation 1). The subroutine generates all possible fragments of length one, and recursively extends the fragments to the right as long as the frequencies of resulting fragments are above the given threshold and the fragments do not exceed the given maximum length. The algorithm is guaranteed to find all frequent fragments, as frequency decreases monotonically when a fragment is extended.

Subroutine 2 ComputeFrequentFragments($\mathcal{H}$, *weight*, *minfr*, *maxlen*)

$\mathcal{F} := \emptyset$;
for all markers i **do**
    for all $a \in A_i$ **do**
        depthFirst($\mathit{frag}(a, i, i)$, $\mathcal{H}$, *weight*, *minfr*, *maxlen*, $\mathcal{F}$);
    end for
end for
return $\mathcal{F}$;

Subroutine 3 DepthFirst($\mathit{frag}(h, i, j)$, $\mathcal{H}$, *weight*, *minfr*, *maxlen*, $\mathcal{F}$)

1: $\mathcal{H}' := \{H \in \mathcal{H} : \mathit{frag}(h, i, j) \text{ matches } H\}$;
2: $\mathcal{F}(\mathit{frag}(h, i, j)) := \sum_{H \in \mathcal{H}'} \mathit{weight}(H)$;
3: **if** $\mathcal{F}(\mathit{frag}(h, i, j)) \geq$ minfr **then**
4:     $\mathcal{F} = \mathcal{F} \cup \{\mathit{frag}(h, i, j)\}$;
5:     **if** $j - i + 1 < \mathit{maxlen}$ **then**
6:         **for all** $a \in A_{j+1}$ **do**
7:             depthFirst($\mathit{frag}(ha, i, j+1)$, $\mathcal{H}'$, *weight*, *minfr*, *maxlen*, $\mathcal{F}$);
8:         **end for**
9:     **end if**
10: **end if**

The frequency of a fragment can be efficiently counted by maintaining, for each fragment, a linked list of haplotypes that match the fragment. This way, each fragment has to be matched only against the haplotypes that match the prefix of the fragment. Moreover, only the last allele of each fragment has to be matched against the haplotypes (line 1 of subroutine DepthFirst).

In the parameter initialization step, a slightly modified version of ComputeFrequentFragments is used

(algorithm not shown). The main difference is that genotypes are used as input instead of haplotypes, and the weighting is done according to Equation 6 instead of weights obtained from the previous haplotype reconstruction step.

Subroutine ReconstructOneGenotype

Pruning The haplotype reconstruction step is implemented by the subroutine ReconstructOneGenotype. The aim is to examine all possible haplotype configurations for each genotype and compute their probabilities. However, exhaustively going through all possible configurations is feasible only when the number of heterozygous markers is small (≈ 20 or less). With more markers, we use a pruning strategy in which the set of possible haplotype configurations is built up marker by marker, starting from a partial configuration containing only the allele pair at the leftmost marker. (Subroutine 4). At each step, all partial configurations are extended with the allele pair at the new marker: for homozygous markers, each configuration is extended with the same allele pair; for heterozygous markers, there are two possible extensions for each configuration, and only the the B most probable configurations are propagated to the next step. In the final step, the $C(\leq B)$ most probable configurations are returned. The approach is greedy; it is not guaranteed that the set of returned configurations consists exactly of the most probable ones. In preliminary experiments it was found that $B = 25$ and $C = 10$ give a reasonable computational efficiency, while increasing the parameters beyond these values does not significantly improve accuracy. These values were used in the experiments.

Subroutine 4 ReconstructOneGenotype$(G, P, \mathcal{F}, B, C)$

$\{C_G^i$ is a set of haplotype fragment pairs compatible with $G(1, i)\}$
$\mathcal{C}_G^1 := \{G(1)\}$;
for all markers $i \in \{2, \ldots, \ell\}$ **do**
  $\mathcal{C}_G^i := \emptyset$;
  $\{a_1, a_2\} := G(i)$;
  for all $\{H_1, H_2\} \in \mathcal{C}_G^{i-1}$ **do**
    $\mathcal{C}_G^i := \mathcal{C}_G^i \cup \big\{\{H_1 a_1, H_2 a_2\}, \{H_1 a_2, H_2 a_1\}\big\}$;
  end for
  Compute probabilities for elements of $\mathcal{C}_G^i$, using P and $\mathcal{F}$;
  Prune away all but the B most probable elements of $\mathcal{C}_G^i$;
end for
return C most probable elements of C_G^ℓ;

Fragment data structure Implementing the haplotype reconstruction step efficiently is crucial for the practical applicability of the algorithm. In this section we describe the data structures and algorithms used

for storing the model parameters and efficiently computing the probabilities defined by the models. We first define some notation for describing the fragment data structure. For all fragments $H(i, j)$, we call fragment $H(i, j-1)$ a *parent* of $H(i, j)$, and similarly call $H(i, j)$ a *child* of $H(i, j-1)$. Moreover, fragment $H(i+1, j)$ is called the *suffix* of fragment $H(i, j)$.

The fragments and their frequencies are stored in a set of tries. In the following, we will associate each fragment with a node of the trie data structure, and interchangeably speak of fragments and nodes of a trie. There is one trie for fragments starting at each marker. The root node of the trie that contains the fragments starting at marker i is denoted by $\mathit{tries}[i]$. The root node represents $\mathit{frag}(\epsilon, i, i-1)$, which is the empty fragment starting at marker i. Each fragment h contains an array *children*; for each allele a, $\mathit{frag}(h, i, j).\mathit{children}[a]$ contains a link to the fragment $\mathit{frag}(ha, i, j+1)$. For example, the root of the trie at marker i, $\mathit{tries}[i].\mathit{children}$, contains links to all fragments of length one at marker i. Each fragment h also contains a link to its parent, denoted $h.\mathit{parent}$.

The frequency of each fragment is stored in the corresponding node, but for compatibility with the previous notation, we will still denote the frequency of $H(i, j)$ by $\mathcal{F}(H(i, j))$. The conditional probability corresponding to any fragment h is obtained as $\mathcal{F}(h)/\mathcal{F}(h.\mathit{parent})$. Finally, each fragment h contains a link to its suffix (contained in the trie of the next marker), denoted as $h.\mathit{suffix}$, while $\mathit{suffixes}(h)$ denotes the set of fragments obtained by recursively following the suffix links starting from h. We define that $\mathit{suffixes}(H(i, j))$ also contains the fragment itself, as well as the empty fragment $\mathit{frag}(\epsilon, j+1, j)$.

Efficiently extending haplotype configurations A crucial operation in Algorithm ReconstructOneGenotype is computing the probabilities of the extended haplotypes based on the probabilities of their prefixes. In the following, we describe how the fragment data structure is used to efficiently implement this for each of the models.

1. Markov model of fixed order: Expressing Equation 1 in terms of fragment frequencies gives the following product:

$$P(H) = \mathcal{F}(H(1, d)) \prod_{i=d+1,\ldots,\ell} \frac{\mathcal{F}(H(i-d, i)}{\mathcal{F}(H(i-d, i-1))}.$$

   The computation breaks down into a product of conditional probabilities, with one multiplication in each extension step. For the fixed-order Markov model extending the probability of a partial haplotype is simple:

$$P\big(H(1, i)\big) = P\big(H(1, i-1)\big) \frac{\mathcal{F}\big(H(i-d, i)\big)}{\mathcal{F}\big(H(i-d, i-1)\big)}.$$

   For each partial haplotype $H(1, i)$, a link to the fragment $H(i-d, i)$ (spanning $d+1$ last markers of

$H(1,i)$) is maintained. The required fragments can be accessed in constant time by alternating between child and suffix links: for each i, $H(i-d,i-1)$ is obtained as $H(i-d-1,i-1).\mathit{suffix}$, from which $H(i-d,i)$ is then obtained as $H(i-d,i-1).\mathit{children}[H(i)]$.

2. Markov model of variable order: The solution for the variable-order Markov model is very similar to the case of fixed-order Markov model. The only difference is that now a link to the longest fragment ending at i, $H(s_i,i)$, is maintained for each partial haplotype $H(1,i)$, instead of the fragment corresponding to the fixed context length, $H(i-d,i)$. $H(s_i,i)$ is obtained from $H(s_{i-1},i-1)$ as $h.\mathit{children}[H(i)]$, where h is the longest suffix of $H(s_{i-1},i-1)$ such that $h.\mathit{children}[H(i)] \neq$ null. When a suffix link is traversed, the index of the leftmost marker in the new fragment is always one larger than in the previous marker. Although multiple suffix links may have to be traversed in a single extension step, the total number of suffix links traversed when reconstructing a single complete haplotype is thus at most equal to the length of the haplotype, and the amortized cost of a single extension step is constant.

3. Segmentation model: For the segmentation model, the probability of a haplotype H is computed as a product of frequencies of fragments that cover H, averaged over all possible segmentations (Equation 4). The summing of the numerator (denoted by $P'(H(1,k)))$ over all possible segmentations, ignoring the normalization factor, can be done with dynamic programming as follows:

$$P'\big(H(1,k)\big) = \sum_{i:s_k,\ldots,k} P\big(H(1,i-1)\big)\mathcal{F}\big(H(i,k)\big)\cdot q,$$

where $s_k = \min\{j : \mathcal{F}\big(H(j,k)\big) \geq \text{minfr}\}$, that is, the first marker of the longest frequent fragment ending at k. This is because each segmentation of H(1,k) contains, as the last fragment, some fragment ending at k, $H(i,k)$, and the sum of probabilities of all segmentations containing $H(i,k)$ is just the sum of the probabilities of all the segmentations up to $(i-1)$, $(P(H(1,i-1)))$, multiplied by the probability of the last fragment, $P(H(i,k))$.

To compute the the sum, the probabilities of partial haplotypes $P'\big(H(1,i)\big)$ and corresponding fragment frequencies $\mathcal{F}\big(H(i,k)\big)$ are needed for all $i : s_k \leq i \leq k$. The probabilities of partial haplotypes are stored by using a backwards-linked list: each partial haplotype $H(1,i)$ contains a link to its prefix, $H(1,i-1)$. As the set of fragments compatible with H and ending at marker k is the same as the set of suffixes of $H(s_k,k)$, the fragments can be enumerated by following the suffix links from $H(s_k,k)$. Thus the probability of each partial haplotype $H(1,k)$ can be computed in time

proportional to the length of the longest compatible fragment that ends in marker k.

Computational complexity

The time complexity of the method is linear in all important parameters (see section Complexity analysis below).

The time complexity of the haplotype reconstruction step is $\mathcal{O}(|\mathcal{G}|\, B\ell)$ for the Markov models, and $\mathcal{O}(|\mathcal{G}|\, \ell_{fr} B\ell)$ for the segmentation model, where ℓ_{fr} is the length of the longest frequent fragment. In other words, it is linear in the number of genotypes ($|\mathcal{G}|$), the number of markers (ℓ), and the number of partial configurations maintained in the reconstruction step (parameter B).

The time complexity of the parameter estimation step is $|A| \cdot 2C \cdot |\mathcal{G}| \cdot |\mathcal{F}| \cdot fr_{avg}$, where fr_{avg} is the average frequency of frequent fragments and $|A|$ is the number of different alleles.

The complexity of performing a single iteration of the algorithm is thus linear in the number of genotypes ($|\mathcal{G}|$), the number of best configurations used in the parameter estimation step (parameter C), the number of different alleles ($|A|$), the total number of frequent fragments ($|\mathcal{F}|$) and the average (normalized) frequency of frequent fragments (fr_{avg}). In our test cases the algorithm has typically converged in 10 to 20 iterations.

Handling of missing data

Practically all real genotype data sets have some missing values, due to limitations of laboratory methods used to determine the alleles. The approach taken by most existing haplotyping methods is to impute alleles (either all possible alleles or only the most probable ones) in the place of the missing ones when reconstructing a haplotype. The approach in this paper is in contrast with the previous approaches. The pattern language of haplotype fragments is extended so that also fragments with missing alleles (wild-card characters) are included in the set of model parameters. When reconstructing a haplotype configuration, the missing marker of a genotype will be missing also in the reconstructed haplotype configurations. In the haplotype reconstruction step, the missing alleles are treated equivalently with any other allele; a missing allele in a fragment only matches a missing allele in a haplotype. This means that no changes need to be done to the haplotype reconstruction step to account for missing data.

In the parameter estimation step, the frequency of a fragment with a missing allele is obtained as the sum of frequencies of the matching full haplotype fragments. A problem that has to be solved when estimating frequencies of fragments in the presence of missing data is how a haplotype containing missing data

contributes to the weight of a matching fragment that has a non-missing allele in at least one marker that is missing in the haplotype. We solve this by dividing the probability mass of a haplotype over all the fragments obtainable by imputing all possible alleles in the missing marker, weighted by the allele frequencies at the missing markers:

$$\mathcal{F}\big(\mathit{frag}(h,i,j)\big) = \frac{1}{|\mathcal{H}|} \sum_{H\in\mathcal{H},H \text{ matches } \mathit{frag}(h,i,j)} \prod_{k:G(k)=*} \mathcal{F}\big(H(k)\big) \text{ weight}(H),$$

where $\mathcal{F}(H(k))$ is the frequency of allele $H(k)$ in marker k, and * denotes a missing allele. The required allele frequencies can be obtained easily as a preprocessing step. This weighting scheme can also be straightforwardly adapted to work with the parameter initialization step (Equation 6), which works directly on the genotypes.

Possible improvements to the methods

For reconstructing the haplotypes on the last iteration of the algorithm, it could be useful to develop a pruning strategy that is guaranteed to find the configuration that has the maximal probability (according to the learned model), instead of the current greedy approach. For the fixed-order Markov chain, this should be a relatively simple task, using an adaptation of the well known Viterbi algorithm (see e.g. [3]). It is however not clear whether the approach is adaptable to the VMM and segmentation models. In the intermediate iterations of the algorithm, it is not sufficient to only find the single best haplotype pair; instead, a sample from the set of consistent haplotype pairs is needed.

The current algorithm is somewhat mixed in its representation of the data: the fragments employed by the models describe local regularities in the data, while complete haplotypes are produced at each reconstruction step. Only a fixed number of haplotype configurations of each genotype is propagated to the parameter estimation step. When the number of markers is very large, this may cause a major fraction of the uncertainty in phase relationships between neighboring markers to be ignored since a list of B haplotype configurations can only represent alternative phases in $log(B)$ positions. A potential improvement on this could be to use an adaptation of the Baum-Welch algorithm (see e.g. [3]) instead of the current approach based on the EM algorithm. Instead of reconstructing complete haplotypes in the intermediate iterations of the algorithm, only the phase relationships between nearby markers would be estimated. In the case of a fixed-order Markov model of order d, this would in practice mean estimating probabilities of partial haplotype configurations of length $d+1$. In the final iteration, these conditional probabilities could then be combined into the most likely complete haplotype configuration, using the

equivalent of the Viterbi algorithm. It is, however, unclear whether this approach would be extendable to the VMM and segmentation models.

2 Proof of convergence

We will prove the local convergence of HaploRec for the Markov model of order d. More specifically, we consider the HaploRec algorithm without the pruning phase.

Let $\mathcal{G} = \{G_1, \ldots, G_N\}$ be the set of observed genotypes consisting of n alleles. The complete data for $\mathcal{G}$ consists of $2N$ haplotypes H_i and $\overline{H}_i$, for $i \in [1, N]$, each of them given by a sequence of haplotype fragments, i.e., $(1a_1 \cdots da_d, 1a_1 \cdots (d+1)a_{d+1}, 2a_2 \cdots (d+2)a_{d+2}, \ldots, (n-d-1)a_{n-d-1} \cdots na_n)$, where $ia_i \cdots ja_j$ denotes the fragment consisting of the alleles a_k at position k, for $k \in [i, j]$.

The parameters which need to be estimated are (1) the probabilities $\Theta_{1a_1 \cdots da_d}$ that alleles $a_1 \cdots a_d$ appear on the first d positions; and (2) the conditional probability $\Theta_{ia_i \cdots (i+d)a_{i+d}}$ that alleles $a_i \cdots a_{i+d}$ appear on positions $i, \ldots, i+d+1$, given that alleles $a_i \cdots a_{i+d-1}$ appear on position $i, \ldots, i+d-1$. We need to estimate these probabilities for all such position intervals of length $d+1$ (and d for the first interval) and possible alleles. We will abbreviate the set of all these parameters by Θ. We omit the estimation for the parameters $\Theta_{1a_1 \cdots da_d}$ since they can be obtained from $\sum_a \Theta_{1a_1 \cdots da_d(d+1)a}$.

Suppose that we have estimates for the parameters in Θ, then we can define for each genotype G_i, for $i \in [1, N]$, the function $f_i(G_i, H, \overline{H} \mid \Theta)$ which is equal to 0 in case H and $\overline{H}$ are not compatible with G_i; is equal to

$$2\Theta_{1a_1 \cdots da_d} \prod_{i=1}^{n-d} \Theta_{ia_i \cdots (i+d)a_{i+d}} \Theta_{1\bar{a}_1 \cdots d\bar{a}_d} \prod_{j=1}^{n-d} \Theta_{j\bar{a}_i \cdots (j+d)\bar{a}_{j+d}}$$

in case $H \neq \overline{H}$; and equal to $(\Theta_{1a_1 \cdots da_d} \prod_{i=1}^{n-d} \Theta_{ia_i \cdots (i+d)a_{i+d}})^2$ otherwise. Here, the a_i's denote alleles in the fragments in H; similarly the $\bar{a}_i$'s denote the alleles in the fragments in $\overline{H}$.

Furthermore, for each genotype $G_i \in \mathcal{G}$, we define the probability distribution

$$P(H, \overline{H} | G_i) = \frac{f_i(G, H, \overline{H})}{\sum_{H, \overline{H}} f_i(G, H, \overline{H})} \tag{7}$$

We then proceed as in the standard EM algorithm by defining the expectation function Q and obtain the update rules for the parameters in Θ by maximizing Q through the computation of the partial derivatives. Hence, first we define the function

$$Q = \sum_{i=1}^{N} \sum_{(G_i, H, \overline{H})} P(H, \overline{H} | G_i) \log(f_i(G, H, \overline{H} \mid \Theta_0)) - \sum_{i=1}^{n} \lambda_i(\sum_{a_i \cdots a_{i+d}} ((\Theta_{ia_i \cdots (i+d)a_{i+d}})_0 - 1)),$$

in which we include Lagrangian multipliers λ_i to force the parameters in Θ to sum up to one. Note that Q involves two sets of parameters Θ (i.e., the current set of parameters, implicitly present in P) and Θ_0 (i.e., the new parameters to be estimated).

We then maximize Q by taking the partial derivatives in Q with respect to the parameters in Θ_0 and λ_i, for $i \in [1, n]$, and equating the obtained formulas to zero. A standard calculation shows that Q is maximized by choosing new values for parameters in Θ as follows:

$$\Theta_{ia_i\cdots(i+d)a_{i+d}} = \frac{1}{2N}\sum_{i=1}^{N}\sum_{(G_i,H,\overline{H})} P(H,\overline{H}|G_i)\delta_{ia_i\cdots(i+d)a_{i+d},\{H,\bar{H}\}}, \tag{8}$$

where $\delta_h\{H,\overline{H}\} = 0, 1, 2$ depending on whether the fragment h does appear in neither H nor $\overline{H}$; appears only in H or $\overline{H}$; or appears in both. Note that Equations (7) and (8) are exactly the Expectation and Maximization steps performed by HaploRec.

3 Complexity analysis

We analyze the time requirement of the algorithm in two separate parts: the parameter estimation step and the haplotype reconstruction step. Each step is executed once at each iteration. The total complexity is the sum of the complexities of these two steps, multiplied by the number of iterations.

Haplotype reconstruction step The dominating factors of the complexity of the haplotype reconstruction step (subroutine ReconstructOneGenotype) are the number of genotypes ($|\mathcal{G}|$) and number of markers (ℓ). For the segmentation model, the complexity of each haplotype extension operation depends on the length of the matching fragments, which is bounded by the maximum fragment length (ℓ_{fr}). Parameter B governs the number of best partial haplotype configurations that are maintained in memory during the haplotype reconstruction step. Recall that $|G(i)| \in \{1, 2\}$ for all i: at each marker, each of the B previous configurations is extended with 1 (in the case of a homozygous marker) or 2 (heterozygous marker) partial configurations of length 1. In the case of a heterozygous marker, the most improbable half of the new configurations is pruned away. At most $2B$ extension operations are done at each step, and each operation involves extending both haplotypes of a partial configuration. There are thus in total $4B$ haplotype extension operations. For the Markov models, each extension operation takes constant time; for the segmentation model it takes time linear in the length of the longest frequent fragment ending at the new marker. Reconstructing one genotype thus uses $\mathcal{O}(B\ell)$ time for the Markov models, and $\mathcal{O}(\ell_{fr}B\ell)$ time for the segmentation model, where ℓ_{fr} is the length of the longest frequent fragment. Consequently, the total complexity of the haplotype reconstruction step is $\mathcal{O}(|\mathcal{G}|B\ell)$ for the Markov models, and $\mathcal{O}(|\mathcal{G}|\ell_{fr}B\ell)$ for

the segmentation model.

Parameter estimation step The main parameters determining the time complexity of the parameter estimation step (subroutine ComputeFrequentFragments) are the number of genotypes ($|\mathcal{G}|$), number of frequent fragments ($|\mathcal{F}|$) and parameter C, which gives the number of best configurations for each haplotype that are considered in the fragment search.

For a Markov model of a fixed degree d, $|\mathcal{F}|$ is bounded by$\sum_{s<\ell-d+1}\prod_{s<i<s+d-1}|A_i|$, which can be expressed more simply as $(\ell-d+1)(d+1)^{|A|}$ when $A_i \leq A$ for all i (i.e. when A bounds the number of alleles in all markers). For the other proposed models, the set of fragments is restricted by a frequency threshold; there is no upper limit to the length of fragments, besides the length of the marker map, and no upper limit for $|\mathcal{F}|$ can be derived easily; therefore, $|\mathcal{F}|$ is an constant term in the complexity.

Consider now the number of times each fragment $h \in \mathcal{F}$ has to be matched with a haplotype (line 2 of subroutine DepthFirst). If we denote the set of haplotypes matching a fragment $h \in \mathcal{F}$ by $haplos(h)$, the number of operations needed for h is $|haplos(h.parent)|$.

It is difficult to characterize the number of haplotypes matching each fragment. A trivial upper bound for $haplos(h)$ is $|\mathcal{H}| = 2|\mathcal{G}|C$, the total number of haplotypes generated by the haplotype reconstruction step of algorithm HaploRec. If we make the simplifying assumption that all the haplotypes in $|\mathcal{H}|$ have the same weight, $\frac{1}{2|\mathcal{G}|C}$, then $\mathcal{F}(h) = |haplos(h)| \cdot \frac{1}{2|\mathcal{G}|C}$, which gives $|haplos(h)| = \mathcal{F}(h) \cdot 2C|\mathcal{G}|$. For each frequent fragment h, $|A|$ children are generated and matched against all the haplotypes in $haplos(h)$. The number of matching operations performed for each frequent fragment is thus $|A| \cdot |haplos(h)|$. The total number of matching operations performed by the algorithm is

$$\begin{aligned}\sum_{h\in\mathcal{F}}|A|\cdot|haplos(h)| &= \sum_{h\in\mathcal{F}}|A|\mathcal{F}(h)2C\cdot|\mathcal{G}| = \\ |A|\cdot 2C\cdot|\mathcal{G}|\sum_{h\in\mathcal{F}}\mathcal{F}(h) &= |A|\cdot 2C\cdot|\mathcal{G}|\cdot|\mathcal{F}|\cdot fr_{avg},\end{aligned}$$

where fr_{avg} is the average frequency of frequent fragments.

Under the assumption of uniform weights of the haplotypes, the complexity of the fragment search algorithm is thus linear in the number of genotypes ($|\mathcal{G}|$), parameter C, the number of different alleles ($|A|$), the total number of frequent fragments ($|\mathcal{F}|$) and the average (normalized) frequency of frequent fragments (fr_{avg}). It is not clear how large fr_{avg} will be in practice; in any case, the minimum frequency threshold gives a lower bound for fr_{avg}, and the average value will probably be close to the lower bound.

Performing a similar analysis without the assumption of uniform weights for the input haplotypes is

currently an open problem; it is possible that a very similar complexity could be obtained also without the assumption, by using average-case analysis.

References

1. Dempster AP, Laird NM, Rubin DB: **Maximum likelihood from incomplete data via the EM algorithm**. *J. R. Stat. Soc* 1977, **39**:1–38.
2. Excoffier L, Slatkin M: **Maximum-Likelihood estimation of molecular haplotype frequencies in a diploid population**. *Molecular Biological Evolution* 1995, **12**(5):921–927.
3. Rabiner LR, Juang BH: **An Introduction to Hidden Markov Models**. *IEEE ASSP Magazine* 1986, **3**:4 – 16.
