# Supplementary material for: HaploRec: efficient and accurate large-scale reconstruction of haplotypes
Source: BMC Bioinformatics. 2006 Dec 22;7:542. doi: 10.1186/1471-2105-7-542 (PMC1766938; doi:10.1186/1471-2105-7-542)
Supplement: Additional file 2 — Example results from the 10 data replicates. [file 1471-2105-7-542-S2.pdf]

Additional file 2: Example results from the 10 data replicates.

In the article, we report average results over 10 independently simulated data sets, as described in the body of the text. Here we provide example results from the individual replicates, to illustrate the amount of variance between data sets on one hand, and between relative performances of the tested methods, in particular.

Each of the 10 figures below illustrates the relative switch accuracies of 7 methods on one data set each, as a function of the number of genotypes (cf. Figure 5 in the article which shows the average accuracies for each method over these 10 replicates).

While there is some variance in the accuracies between replicates, the relative performances of the tested methods are extremely similar across all replicates. In particular, for sample sizes (number of genotypes) of 200 or more, the relative results are almost identical; in our other experiments we used a sample size of 1000 genotypes.

The conclusions drawn in the paper would be the same if any single replicate would have been used. By using 10 of them we believe that random effects are well controlled. Running the experiments of the paper on the 10 replicates took about 170 days of CPU time.

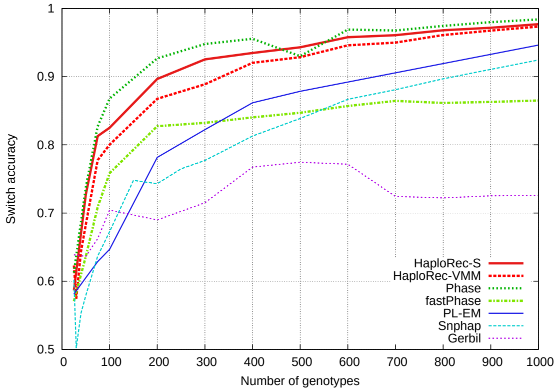

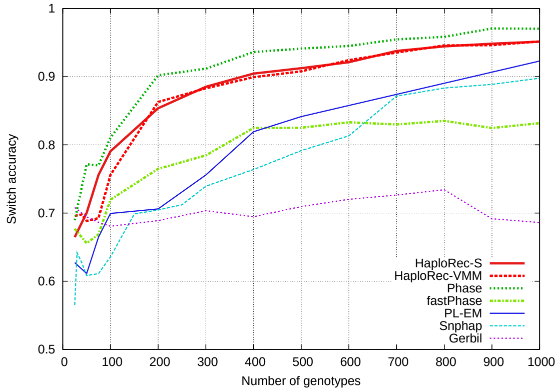

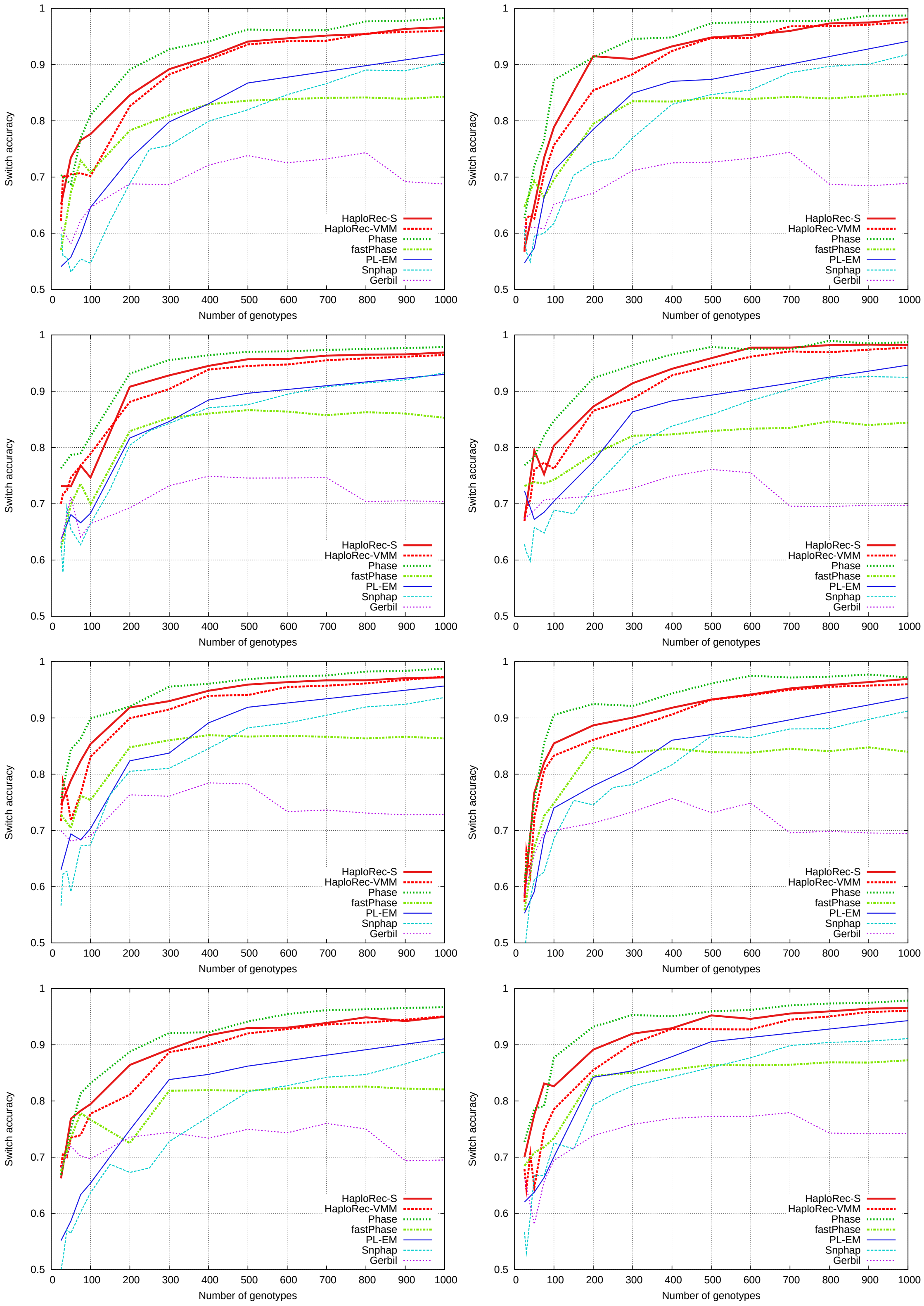
Switch accuracy
Number of genotypes
HaploRec-S
HaploRec-VMM
Phase
fastPhase
PL-EM
Snphap
Gerbil
